# Lattice-hydrogen cycling mechanism enables pH-universal hydrogen evolution at ampere-level current densities

**DOI:** 10.1038/s41467-025-65909-3

**Published:** 2025-12-03

**Authors:** Yan Zhang, Biao Feng, Jingyi Tian, Shiqi Zhou, Changkai Zhou, Yiqun Chen, Xiaoli Xia, Xizhang Wang, Lijun Yang, Luming Peng, Qiang Wu, Hongwen Huang, Zheng Hu

**Affiliations:** State Key Laboratory of Coordination Chemistry, Key Laboratory of Mesoscopic Chemistry of MOE, School of Chemistry and Chemical Engineering, Nanjing University, Nanjing, Jiangsu 210023 P. R. China

**Keywords:** Electrocatalysis, Energy, Electrocatalysis, Hydrogen energy

## Abstract

Controllable supply of hydrogen intermediate across a wide pH range is crucial for electroreduction reactions, but is hindered by pH-dependent hydrogen species formation on conventional catalysts. We report a lattice-hydrogen cycling mechanism that dissociates hydrogen intermediate availability from electrolyte pH. By integrating proton-blocking Ru with thermally-hydrogenated H_x_WO_3_, we create a dynamic hydrogen reservoir, enabling efficient hydrogen supply. In-situ Raman spectroscopy, isotopic labeling, and theoretical simulations reveal the lattice hydrogen in H_x_WO_3_ migrates swiftly to Ru active sites via low-energy-barrier pathways, while consumed hydrogen is spontaneously replenished via proton adsorption (acidic) or water dissociation (alkaline/neutral). Consequently, this catalyst achieves a competitive pH-universal performance for hydrogen evolution reaction, with low overpotentials (125 mV acidic, 142 mV alkaline, 219 mV neutral @1 A cm^-2^) alongside 500-hour stability.

## Abstract (web summary)

Controllable supply of hydrogen during electroreduction reactions on conventional catalysts is hindered by pH-dependent hydrogen species formation. Here the authors present a lattice-hydrogen cycling mechanism that decouples hydrogen intermediate availability from electrolyte pH.

## Introduction

The transition towards sustainable energy and chemical production necessitates innovative strategies to decouple human development from fossil fuel dependence^1^. Electrochemical hydrogen-involving reactions, such as the hydrogen evolution reaction (HER), CO_2_ reduction (CO_2_RR), and nitrogen reduction reaction (NRR), serve as critical bridges connecting renewable energy to sustainable fuel and chemical production^2,3^. In these processes, the kinetics of active hydrogen (H^*^) formation plays an important role in modulating the reaction pathways and reaction kinetics^2,4,5^. It is well established that the formation of H^*^ is highly dependent on the pH of the electrolyte. In acidic media, the abundance of protons (H⁺) facilitates the direct proton-electron coupling (H^+^ + e⁻ →H^*^), while in neutral/alkaline conditions, water dissociation (H_2_O + e⁻ →H^*^ + OH^⁻^) becomes a necessary step, introducing an additional energy barrier^6^. This fundamental dichotomy leads to the severe kinetic penalties in neutral/alkaline environments. As an example, the 2–3 order-of-magnitude drop in HER activity for Pt is observed when transitioning from acidic to alkaline electrolytes^7^. In addition, for renewable electricity-driven electrochemical hydrogen-involving reactions (HER, CO_2_RR, NRR, etc.), the intermittent and fluctuating electricity output induces variations in the current density of practical electrochemical devices, which in turn causes the pH oscillations at the electrified interface under operating conditions^8^. In this context, enabling the controllable and efficient supply of H^*^ in all-pH electrolytes to overcome pH-imposed limitations is of both fundamental and industrial significance.

Focusing on the HER, the local pH value on the catalyst surface often experiences significant changes, especially in direct seawater electrolysis. For instance, the local pH variations exceeding 2 pH units were detected even in strong buffer electrolytes at a moderate current density of −30 mA cm^-2^. The fluctuations will become more pronounced at ampere-level current density, which greatly affect HER performance^9^. One conventional strategy for designing pH-universal catalysts is to integrate an oxyphilic component with a catalytically active component, where the oxyphilic component typically enhances water dissociation, thereby facilitating the efficient supply of H^*^^10,11^. Using this strategy, numerous pH-universal HER electrocatalysts have been reported (Supplementary Tables 1, 2). Although a few of them have shown HER overpotentials less than 30 mV at a benchmark current density of 10 mA cm^-2^, the pH-universal catalysts with low overpotentials ( < 250 mV) at industrial-current density ( ≥ 1 A cm^-2^) in all-pH electrolytes have not yet been realized to date^12^, due to the difficult creation of a densely atomically mixed interface to form H^*^.

As we know, for proton-blocking metal catalysts like Ru, Pt and Ir, the proton reduction and HER occur on the surface. The shift in HER mechanism with local pH restricts the development of pH-universal catalysts^13,14^. In contrast, for non-proton-blocking materials such as certain transition oxides (WO_3_, MoO_3_, etc.), proton can be electrochemically inserted into the crystal lattice in all-pH electrolytes, forming a “hydrogen reservoir”^15–17^. It is anticipated that if rapid proton migration pathways can be established from non-proton-blocking support to proton-blocking metal catalysts, the impact of the pH value on the H^*^ formation can be mitigated or even eliminated, thereby achieving a competitive pH-universal HER performance. With this consideration, we construct a HER catalyst by loading the proton-blocking Ru nanoparticles (NPs) onto the non-proton-blocking H_x_WO_3_ nanoneedles (NN) obtained by thermal hydrogenation of WO_3_ NN, denoted as Ru-H_x_WO_3_ NN. Combining the in-situ experimental results with theoretical simulations, we demonstrate a lattice-hydrogen cycling mechanism: the H_x_WO_3_ NN with abundant lattice hydrogen functions as a “hydrogen reservoir” that can efficiently supply hydrogen species to the Ru sites via a rapid lattice-hydrogen migration. Meanwhile, the consumed lattice-hydrogen in H_x_WO_3_ NN is spontaneously replenished in all-pH electrolytes through hydrogen adsorption (acidic) or water dissociation (alkaline/neutral). Such a unique mechanism confers the Ru-H_x_WO_3_ NN with a competitive pH-universal HER performance, achieving low overpotentials of 125, 219, and 142 mV at an industrial-level current density of 1 A cm^-2^, along with great durability (500 h@1 A cm^-2^) in 0.5 M H_2_SO_4_, 1 M phosphate buffered solution (PBS), and 1 M KOH, respectively.

## Result

### Structural characterization of Ru-H_x_WO_3_ NN and Ru-WO_3_ NN

**Fig. 1:**
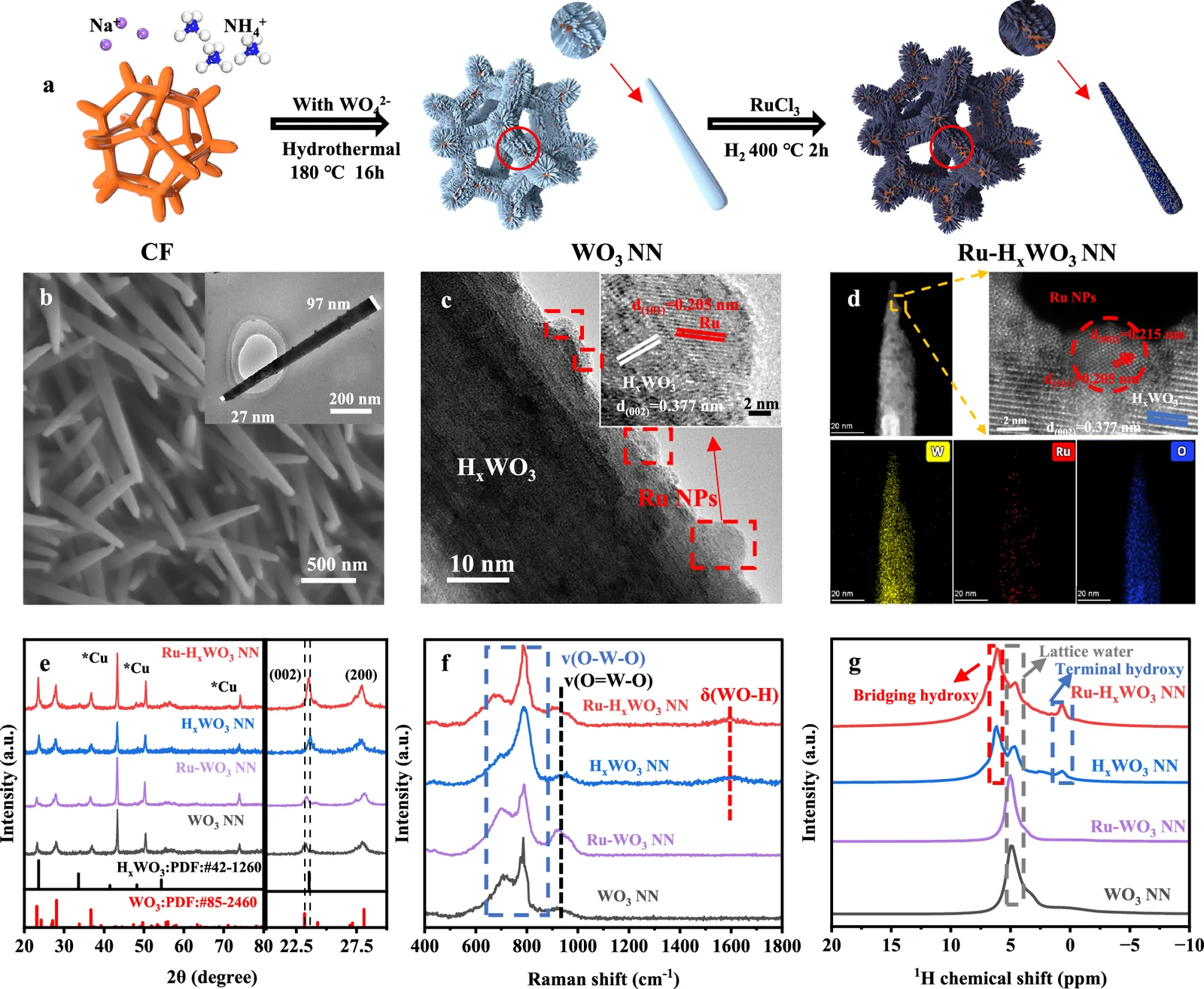
Preparation and characterizations of Ru-H_x_WO_3_ NN. **a** Schematic preparation route. **b,c** SEM (**b**) and HRTEM images (**c**). Insets are the corresponding TEM image and local enlargement. **d** HAADF-STEM image and corresponding EDS elemental mappings of W, Ru, and O. **e** XRD pattern and local enlargement in the range of 20°−30°. **f** Raman spectrum. **g** ^1^H NMR spectrum. Note: The spectra for WO_3_ NN, H_x_WO_3_ NN and Ru-WO_3_ NN are also shown in (**e–g**) for comparison.

The preparation and characterizations of Ru-H_x_WO_3_ NN via thermal-hydrogenation are shown in Fig. 1. For comparison, the synthesis of Ru-WO_3_ NN via NaBH_4_-reduction is also presented (Supplementary Fig. 1, and see details in Methods). Scanning electron microscopy (SEM) and transmission electron microscopy (TEM) observations reveal that WO_3_ NN are densely grown on the surface of copper foam (CF), with typical tip diameter of ~25 nm. Specifically, the WO_3_ NN shows the lattice distances of 0.385 and 0.639 nm for the (002) and (100) planes, respectively. After undergoing H_2_ thermal treatment, the resulting H_x_WO_3_ NN shows a smaller interplanar distance of 0.377 nm for the (002) planes (Supplementary Fig. 2). This phenomenon indicates that hydrogen is inserted into the interlayer galleries of the (002) planes during the thermal reduction process, which in turn leads to an obvious shrinkage of the WO_3_ lattice. CV tests and thermogravimetric analysis (TGA) further confirm the insertion of hydrogen into WO_3_ NN, with x value of ~0.8 in H_x_WO_3_ NN (Supplementary Figs. 3 and 4). It is worth noting that when protons are inserted into WO_3_ to form H_y_WO_3_ by electrochemical method, the typical value of y is ca. 0.5, which is significantly lower than that in the thermally-hydrogenated H_x_WO_3_ NN (Supplementary Fig. 3)^13^.

For Ru-H_x_WO_3_, the nanoneedle morphology is well-preserved, and hemispherical Ru NPs are highly dispersed on the surface of H_x_WO_3_ (Fig. 1b,c and Supplementary Fig. 5). The (101) planes of Ru NPs (with a lattice distance of 0.205 nm) and the (002) facets of H_x_WO_3_ (with a lattice distance of 0.377 nm) form an intersection angle of 45°, showing a good lattice match at the heterogenous interfaces. The high-angle annular dark field scanning transmission microscopy (HAADF-STEM) image of a Ru-H_x_WO_3_ NN, along with the corresponding energy dispersive X-ray spectroscopy (EDS) elemental mapping images, verifies the homogeneous distribution of tungsten (W) and oxygen (O), as well as the dotlike distribution of Ru, which is consistent with the observations from high-resolution transmission electron microscopy (HRTEM) (Fig. 1d). In the case of Ru-WO_3_, the morphology of the small Ru NPs immobilized on the surface of WO_3_ NN is similar to that of Ru-H_x_WO_3_ NN, but the distance between the (002) planes of WO_3_ NN remains at 0.385 nm. This result confirms that the reduction by NaBH_4_ only led to the formation of metallic Ru NPs but did not result in the insertion of hydrogen into WO_3_ (Supplementary Fig. 6).

X-ray diffraction (XRD) analysis shows that, after thermal-hydrogenation, the (002) peak shifts positively from 23.2° to 23.6°. The in situ XRD patterns show that the (002) peak gradually shifts to high angles during the temperature-increasing process, and the position of (002) peak keeps constant in the subsequent cooling process, which confirms the formation of H_x_WO_3_ during the thermal-hydrogenation (Supplementary Fig. 7). This shift also reflects the shrinkage of the (002) lattice after hydrogen insertion, which is in line with the HRTEM observations (Fig. 1e, and Supplementary Figs. 2 and 5). When comparing the Raman spectra of H_x_WO_3_ and Ru-H_x_WO_3_ NN with those of WO_3_ and Ru-WO_3_ NN, a new signal appears at approximately 1580 cm^-1^, which corresponds to the bending vibration band of WO-H bond [δ(WO-H)] (Fig. 1f)^18,19^. The solid-state ^1^H nuclear magnetic resonance (^1^H NMR) spectra of H_x_WO_3_ and Ru-H_x_WO_3_ NN indicate the emergence of bridging hydroxy (H-O_Bridging_, in bulk) and terminal hydroxy (H-O_Terminal_, on surface) groups, accompanied by the weakened signal of lattice water (Fig. 1g)^20^. These results clearly confirm the formation of lattice-hydrogen in H_x_WO_3_ and Ru-H_x_WO_3_ NN through the H_2_ thermal treatment.

**Fig. 2:**
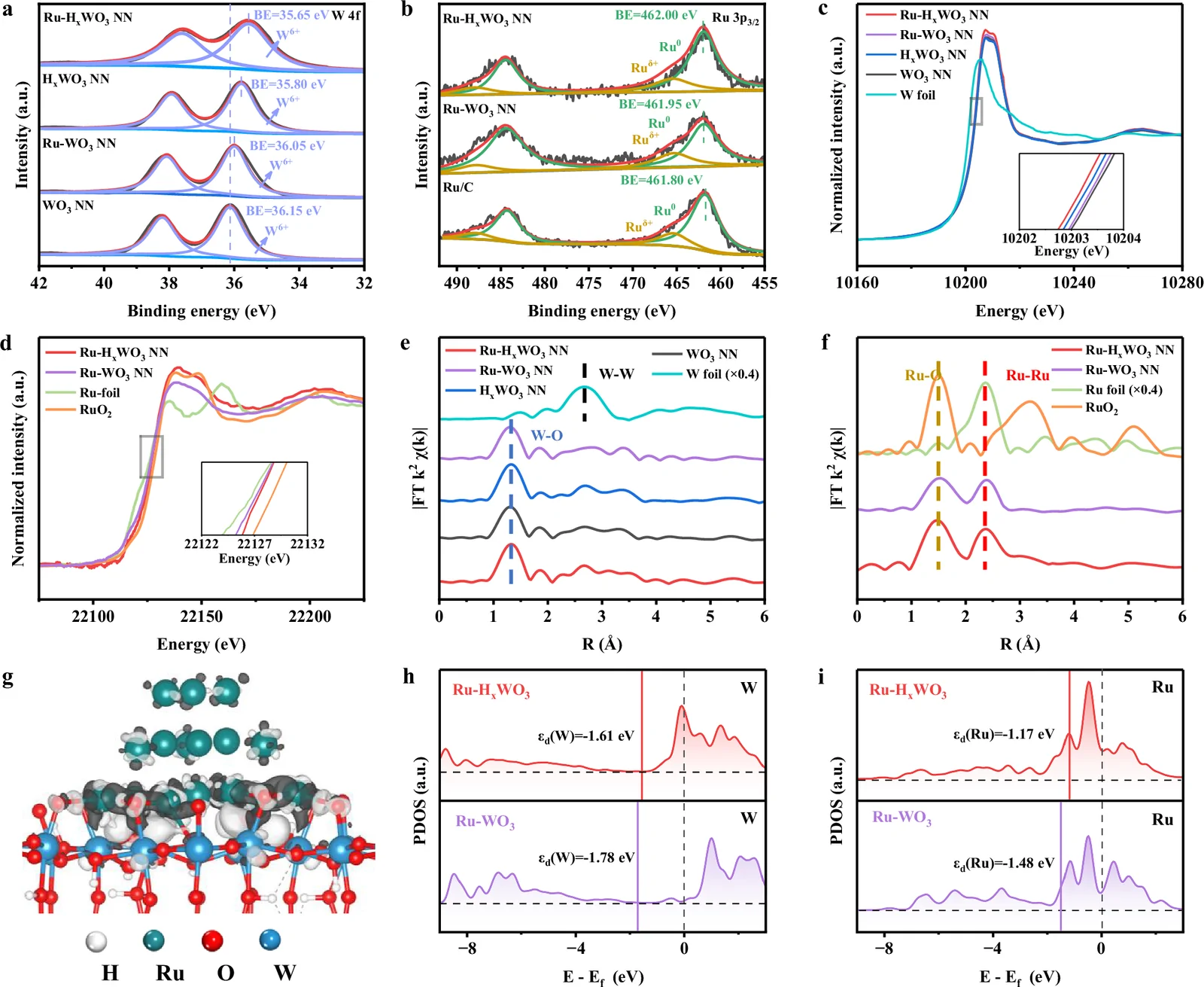
Electronic structure characteristics of Ru-H_x_WO_3_ NN, Ru-WO_3_ NN, WO_3_ NN, and H_x_WO_3_ NN. **a,b** W 4 *f* and Ru 3*p*_*3/2*_ spectra, respectively. For comparison, the spectrum for commercial Ru/C is presented in (**b**). **c,d** W L_3_-edge and Ru K-edge XANES spectra, respectively. For comparison, the spectrum for W foil is presented in (**c**), and those for Ru foil and RuO_2_ are presented in (**d**). The marks in (c) and (d) is local enlargement. **e,f** Corresponding *k*^2^-weighted R-space Fourier transformed EXAFS spectra for W (**e**) and Ru (**f**). **g** Charge difference density. Black and white regions represent electron depletion and accumulation, respectively. The isosurface value is set at 0.01. **h,i** The PDOS curves of 3 *d* orbitals of W (**h**) and Ru (**i**) atoms in Ru-H_x_WO_3_ NN and Ru-WO_3_ NN. The *d*-band centers of the corresponding metal are marked in black font.

The electronic structure characteristics of Ru-H_x_WO_3_ NN, Ru-WO_3_ NN, WO_3_ NN, and H_x_WO_3_ NN are presented in Fig. 2. X-ray photoelectron spectroscopy (XPS) analysis reveals the existence of W^6+^ and Ru^0^ species in Ru-H_x_WO_3_ NN and Ru-WO_3_ NN, with the close Ru contents of 1.21 and 1.26 wt.%, respectively (Fig. 2a, b and Supplementary Table 3). The binding energy (BE) of the W^6+^ species in WO_3_ NN is 36.15 eV. In contrast, the BE of the W^6+^ species negatively shifts to 35.80 eV in H_x_WO_3_ NN due to hydrogen insertion, and further negatively shifts to 35.65 eV in Ru-H_x_WO_3_ NN due to the additional loading of Ru. When only Ru is loaded (i.e., without hydrogen insertion), the corresponding BE slightly negatively shifted to 36.05 eV in Ru-WO_3_ NN^21^ (Fig. 2a). Regarding the Ru 3*p* spectra, when compared with commercial Ru/C, the Ru^0^ signal in Ru-H_x_WO_3_ and Ru-WO_3_ NN shows positive BE shifts of 0.20 and 0.15 eV, respectively^22^ (Fig. 2b). The XPS results reflect an electron transfer from Ru to H_x_WO_3_ NN and WO_3_ NN.

X-ray absorption fine structure (XAFS) spectra were employed to further reveal chemical state of W and Ru (Fig. 2c, d). The W L_3_-edge X-ray absorption near-edge structure (XANES) spectra indicate that the edge energy changes in the order of Ru-H_x_WO_3_ NN <H_x_WO_3_ NN <Ru-WO_3_ NN < WO_3_ NN. The Ru K-edge XANES spectra show that the energy absorption threshold values of Ru-H_x_WO_3_ NN and Ru-WO_3_ NN are slightly higher than that of Ru foil with the former being more positive. These results are in agreement with XPS results (Fig. 2a–d). The XAFS results also suggest a strong interaction between Ru and WO_3_ or H_x_WO_3_ NN, with electron transfer from Ru^0^ to W^6+^. The W *k*^2^-weighted R-space Fourier transform extended X-ray absorption fine structure (FT-EXAFS) spectra for all samples display a main peak around 1.40 Å for the W-O bond, without the appearance of the W-W signal around 2.75 Å (Fig. 2e). The Ru *k*^2^-weighted R-space FT-EXAFS spectra for Ru-H_x_WO_3_ NN and Ru-WO_3_ NN show two peaks around 1.55 and 2.40 Å, which correspond to Ru-O and Ru-Ru bonds, respectively. These results and corresponding theoretical calculations confirm that the Ru NPs bond to the H_x_WO_3_ and WO_3_ NN through Ru-O ionic bonds, rather than Ru-W metallic bonds (Fig. 2f, and Supplementary Fig. 8).

Accordingly, a model of Ru-H_x_WO_3_ was constructed to calculate the work function and charge difference density (Fig. 2g). Density functional theory (DFT) calculations indicate that the work functions of the Ru (101) plane and the H_x_WO_3_ (002) plane are 4.75 and 5.17 eV, respectively. The lower work function of Ru than H_x_WO_3_ suggests a tendency of electron transfer from Ru to H_x_WO_3_. The charge difference density analysis corroborates this result, showing electron accumulation at the H_x_WO_3_ surface and electron depletion at the Ru plane (Fig. 2g). This electron redistribution causes the *d*-band centers of Ru and W in the Ru-H_x_WO_3_ system to shift closer to the Fermi level (E_f_) compared to those in Ru-WO_3_, which can enhance the adsorption stability of key intermediates (e.g., H^*^, H_2_O^*^) on Ru-H_x_WO_3_^23^. Moreover, the higher Fermi level occupancy (E-E_f_ = 0) for Ru and W in Ru-H_x_WO_3_ further facilitates the electron conductivity and HER thereof (Fig. 2h, i)^24^. Overall, compared with Ru-WO_3_ NN, the thermally-hydrogenated H_x_WO_3_ NN induces more electron transfer from Ru to H_x_WO_3_ NN, leading to enhanced electron conductivity and improved intermediate adsorption in Ru-H_x_WO_3_ NN.

### Electrocatalytic HER performances

**Fig. 3:**
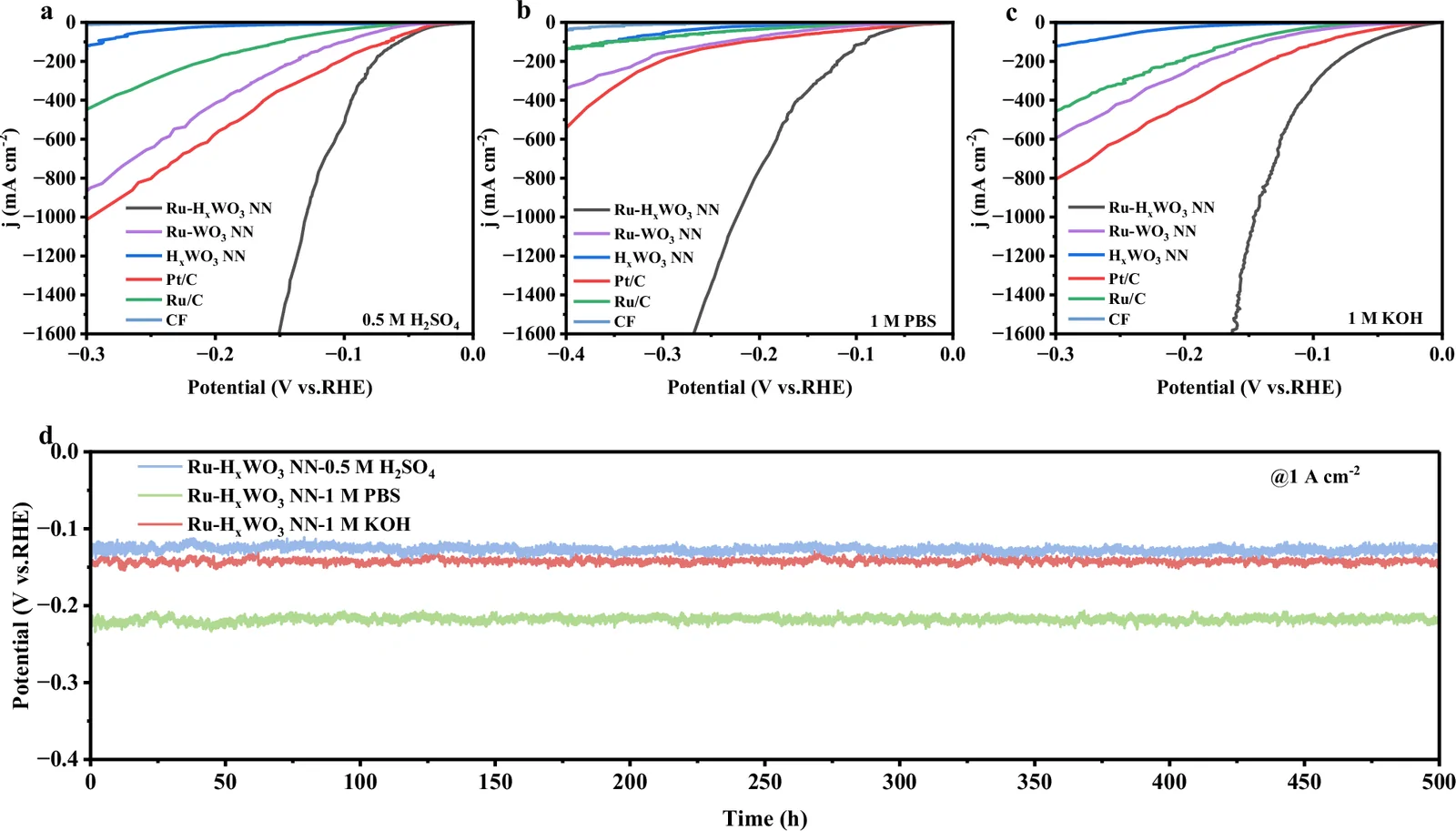
pH-universal HER performances of Ru-H_x_WO_3_ NN, Ru-WO_3_ NN, and H_x_WO_3_ NN in 0.5 M H_2_SO_4_, 1 M PBS and 1 M KOH. **a–c** Polarization curves. **d** CP curves of Ru-H_x_WO_3_ NN at the current density of 1 A cm^-2^ for 500 h. Note: The data of Pt/C, Ru/C and CF in (**a–c**) are presented for comparison.

The HER performances of Ru-H_x_WO_3_ NN, Ru-WO_3_ NN, H_x_WO_3_ NN, along with the benchmark catalysts Pt/C and Ru/C, were systematically examined, as illustrated in Fig. 3. In 0.5 M H₂SO₄, 1 M PBS, and 1 M KOH electrolytes, Ru-H_x_WO_3_ NN exhibits low overpotentials of 12 mV, 28 mV, and 14 mV, respectively, at a current density of 10 mA cm^-2^. These values are significantly lower than those of Pt/C and Ru/C (Fig. 3a–c). In contrast, under the same conditions, Ru-WO_3_ NN displays notably higher overpotentials of 45, 80, and 53 mV, while H_x_WO_3_ NN shows the high overpotentials of 140, 148, and 151 mV, respectively. This result clearly demonstrates the lattice hydrogen in H_x_WO_3_ NN plays a crucial role in the great HER performance of Ru-H_x_WO_3_ NN due to the heterogenous coupling between Ru and H_x_WO_3_ NN (Fig. 3a-c and Supplementary Fig. 9). When normalized by the electrochemical active surface area (ECSA), Ru-H_x_WO_3_ NN also shows the best pH-universal performance among the examined catalysts, indicating its optimal intrinsic activity for the HER (Supplementary Figs. 10 and 11). Notably, Ru-HxWO₃ NN exhibits mass activities at 100 mV that are competitive with common benchmarks (Pt/C and Ru/C), consistent with efficient utilization of noble metals (Supplementary Fig. 12). Electrochemical impedance spectroscopy (EIS) analysis reveals that Ru-H_x_WO_3_ NN has the smallest charge-transfer resistance (R_ct_) among all the catalysts in all-pH electrolytes, reflecting its accelerated HER kinetics (Supplementary Fig. 13).

The Ru-H_x_WO_3_ NN catalyst demonstrates a competitive pH-universal HER performance even at an ampere-level current density of 1 A cm^-2^, with the low overpotentials of 125 mV, 219 mV, and 142 mV in 0.5 M H_2_SO_4_, 1 M PBS and 1 M KOH electrolytes, respectively (Supplementary Fig. 9). Attractively, an extraordinary long-term durability for over 500 h is achieved at 1 A cm^-2^ in all-pH electrolytes, with negligible changes in overpotentials and polarization curves before and after the chronopotentiometry (CP) tests (Fig. 3d and Supplementary Fig. 14). Furthermore, after the durability tests in all-pH electrolytes, the morphology, composition and structure of Ru-H_x_WO_3_ NN remain almost identical to those of the pristine catalyst, indicating its high structural stability and electrocatalytic stability (Supplementary Figs. 15, 16 and Table 4).

Moreover, among the examined catalysts, the Ru-H_x_WO_3_ NN exhibits the lowest Tafel slopes, with values of 28.8, 34.5, and 34.2 mV dec^-1^ in 0.5 M H_2_SO_4_, 1 M PBS and 1 M KOH, respectively. In contrast, the Tafel slopes of Ru-WO_3_ NN in the same electrolytes are 55.8, 86.5, and 82.6 mV dec^-1^, respectively, which are close to those of Ru/C (Supplementary Fig. 17). This result implies that the rate-determining step (RDS) for Ru-H_x_WO_3_ NN is the Tafel process, while those for Ru-WO_3_ NN and Ru/C are the Heyrovsky process^25^. This disparity can be attributed to the significant enhancement effect of the H_x_WO_3_ NN on the HER performance of Ru NPs.

Generally speaking, Ru-H_x_WO_3_ NN exhibits the most competitive pH-universal HER performance among the examined catalysts (Fig. 3d, and Supplementary Table 1 and Fig. 18). Notably, it is also competitive with most HER catalysts in single electrolytes at ampere-level current density (Supplementary Table 2).

### The HER enhancement mechanism of Ru-H_x_WO_3_ NN

#### The HER characteristics of the HₓWO_3_ support

**Fig. 4:**
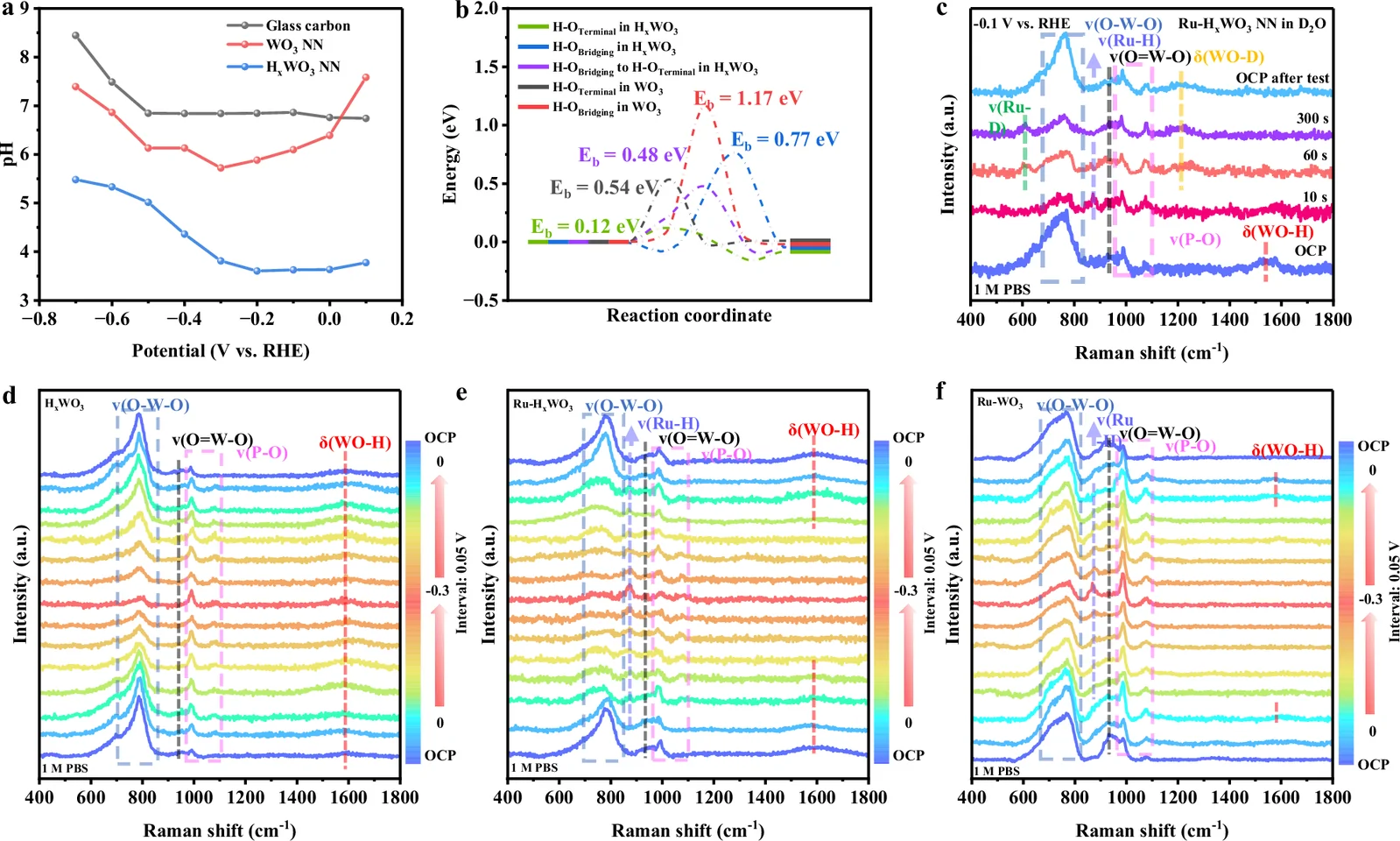
The behavior of lattice-hydrogen in Ru-H_x_WO_3_ NN and H_x_WO_3_ NN. **a** Local pH values on the surfaces of H_x_WO_3_ NN, WO_3_ NN and GC at different potentials in 0.1 M PBS with pH of 7.02. **b** Calculated energy barrier diagram for H migration. **c** In situ Raman spectra of Ru-H_x_WO_3_ NN at -0.1 V in 1 M PBS D_2_O solution at different stages of HER. **d–f** In situ Raman spectra of H_x_WO_3_ (**d**), Ru-H_x_WO_3_ (**e**) and Ru-WO_3_ (**f**) NN in 1 M PBS solution at different potentials. Note: The Raman signals at ~985 and ~1075 cm^-1^ are attributed to the symmetric P-O stretching mode of PO_4_^3-^ from the electrolyte^42^.

We first investigated the characteristics of the HER of the HₓWO_3_ support itself (x = 0.88 ~ 0.97). The rotating ring-disk electrode (RRDE) technique was employed to quantitatively monitor the local pH on the H_x_WO_3_ NN surface at different potentials in 0.1 M PBS solution (pH=7.02) (Supplementary Figs. 19 and 20)^26^. The results were compared with those of WO_3_ NN and glassy carbon (GC). It was found that the pH on GC remained nearly constant at ~7.0 within the potential range of 0.1 ~ -0.5 V. In contrast, the local pH on the surface of H_x_WO_3_ NN was approximately 3.7 within 0.1 ~ -0.2 V, indicating the presence of a local acid-like microenvironment due to the thermally-inserted hydrogen in H_x_WO_3_ NN (Fig. 1f,g)^17,27^. The local pH on the surface of WO_3_ NN decreased from 7.58 (weak basicity) to 5.72 (weak acidity) as the potential shifted negatively from 0.1 V to -0.3 V, due to the electrochemical insertion of hydrogen into WO_3_ to form H_y_WO_3_ (y = ~0.5) (Fig. 4a)^13^. The lower local pH on H_x_WO_3_ NN than on WO_3_ NN indicates that thermal H insertion can result in a higher degree of hydrogenation of WO_3_ than electrochemical H insertion. This situation leads to a much higher HER performance of Ru-H_x_WO_3_ NN than Ru-WO_3_ NN (Supplementary Fig. 21). As the potential is further shifted negatively, the local pH values gradually increase due to the consumption of H species during the HER. For both H_x_WO_3_ NN and WO_3_ NN, the lattice-H can migrate between the adjacent oxygen sites. The energy barriers for this migration are much lower in H_x_WO_3_ than in WO_3_, indicating the enhanced lattice-H migration kinetics in H_x_WO_3_ (Fig. 4b and Supplementary Fig. 22). After removing the bias, the pH on H_x_WO_3_ NN can return to a low value (Supplementary Fig. 20e). This result reflects that the lattice-H species in H_x_WO_3_ can be spontaneously replenished to go back to the local acidity. Similar phenomena were also observed in H_2_SO_4_ and KOH electrolytes, demonstrating the formation of local acid-like microenvironment on the H_x_WO_3_ surface under all-pH conditions (Supplementary Figs. S19 and S20).

#### The enhanced HER activity of Ru-H_x_WO_3_ NN

To verify the participation of lattice-H in the HER of H_x_WO_3_ NN, in situ Raman spectroscopy was used to monitor the HER process of Ru-H_x_WO_3_ NN in 1 M PBS isotope deuteroxide (D_2_O) solution at -0.1 V (Fig. 4c). At the open circuit potential (OCP), the δ(WO-H) peak at ~1580 cm^-1^ was observed as expected. On applying the potential of -0.1 V (10 s), the stretching vibration peak of ν(Ru-H) at ~872 cm^-1^ appeared^28^, accompanied by the weakening of the δ(WO-H) peak. After 60 s’ testing, only trace of the ν(Ru-H) and δ(WO-H) peaks remained, and two new peaks of ν(Ru-D) at ~600 cm^-1^ and δ(WO-D) at ~1200 cm^-1^ were observed^19^. At 300 s, the ν(Ru-H) and δ(WO-H) peaks completely disappeared, leaving the ν(Ru-D) and δ(WO-D) signals. It is seen, during this process, the δ(WO-H)/ν(Ru-H) is gradually replaced by the δ(WO-D)/ν(Ru-D). When returning OCP condition, only δ(WO-D) signal could be detected. Similar process was also observed in 1 M KOH electrolyte with D_2_O solvent (Supplementary Fig. 23). These results confirm the dynamic migration of lattice-H from the H_x_WO_3_ support to Ru for the HER, and the replenishment of lattice-D through the electrochemical insertion of deuterium. In addition, the kinetic isotope effect (KIE) was examined in either 1 M PBS or 1 M KOH electrolyte with H_2_O or D_2_O solvent^29^. The KIE values (*J*_H2O_/*J*_D2O_) remained constant for Ru-WO_3_ NN, while increased rapidly for Ru-H_x_WO_3_ NN, reflecting the migration and reconstruction of lattice-H/D during the HER for Ru-H_x_WO_3_ NN (Supplementary Fig. 24). Therefore, the isotope experiment results clearly prove the involvement of pre-inserted lattice-H and in situ electrochemical replenishment of lattice-H/D during the HER of Ru-H_x_WO_3_ NN.

To gain a deeper understanding of the enhanced HER activity of Ru-H_x_WO_3_ NN catalyst, in situ Raman spectra were recorded. The potential was increased negatively from 0 to -0.3 V and then returned to 0 V, with a potential interval of 0.05 V. In 1 M PBS solution, for the H_x_WO_3_ NN support, the ν(W-O) peaks at 698, 790, and 920 cm^-1^ became weaker/stronger as the potential was increased negatively and then recovered, respectively, while the δ(WO-H) peak at 1580 cm^-1^ showed an opposite trend of change^18,30^ (Fig. 4d). This result indicates that the lattice-H concentration in H_x_WO_3_ can be further regulated by the electrochemical process^17^. In contrast, for the Ru-H_x_WO_3_ NN, both the ν(W-O) and δ(WO-H) peaks of H_x_WO_3_ became weaker/stronger as the potential was increased negatively and then recovered, respectively. Meanwhile, a new ν(Ru-H) peak emerged, and became stronger/weaker as the potential was increased negatively and then recovered, correspondingly (Fig. 4e). These results clearly indicate the lattice-H in H_x_WO_3_ NN [δ(WO-H)] can dynamically migrate to the Ru NPs [ν(Ru-H)] under the applied potential, meanwhile the lattice-H can be in situ replenished electrochemically. The weakening of ν(W-O) peaks is directly associated with the migration of lattice-H via hopping on lattice oxygen (Fig. 4b). As a comparison, for Ru-WO_3_ without pre-inserted lattice-H, a trace of the δ(WO–H) and ν(Ru-H) peaks could be observed at -0.05 V, which is associated to the electrochemical insertion of hydrogen into WO_3_ and its subsequent migration to the Ru NPs. Then, the Raman spectra of Ru-WO_3_ show a similar evolution to those of Ru-H_x_WO_3_ NN (Fig. 4f). The residual intensity of ν(W-O) peaks at -0.3 V is much stronger in Ru-WO_3_ than in Ru-H_x_WO_3_. These results indicate that, compared to Ru-H_x_WO_3_, Ru-WO_3_ has a lower lattice-H concentration from electrochemical H insertion and less dynamic migration of lattice-H to Ru. This situation leads to a higher intensity of the ν(Ru-H) signal in Ru-H_x_WO_3_ than in Ru-WO_3_, i.e., higher H^*^ coverage on Ru for the former (Supplementary Fig. 25). Similar results were also obtained in H_2_SO_4_ and KOH electrolytes (Supplementary Figs. 25 and 26). The higher H^*^ coverage on Ru in Ru-H_x_WO_3_ than in Ru-WO_3_ in all-pH electrolytes is responsible for its enhanced pH-universal HER performance. Our in situ EIS study on the H adsorption behaviors at different overpotentials also support this conclusion (Supplementary Figs. 27–29 and Tables 5–7)^31,32^.

### The cycling of hydrogen migration, evolution, and replenishment

**Fig. 5:**
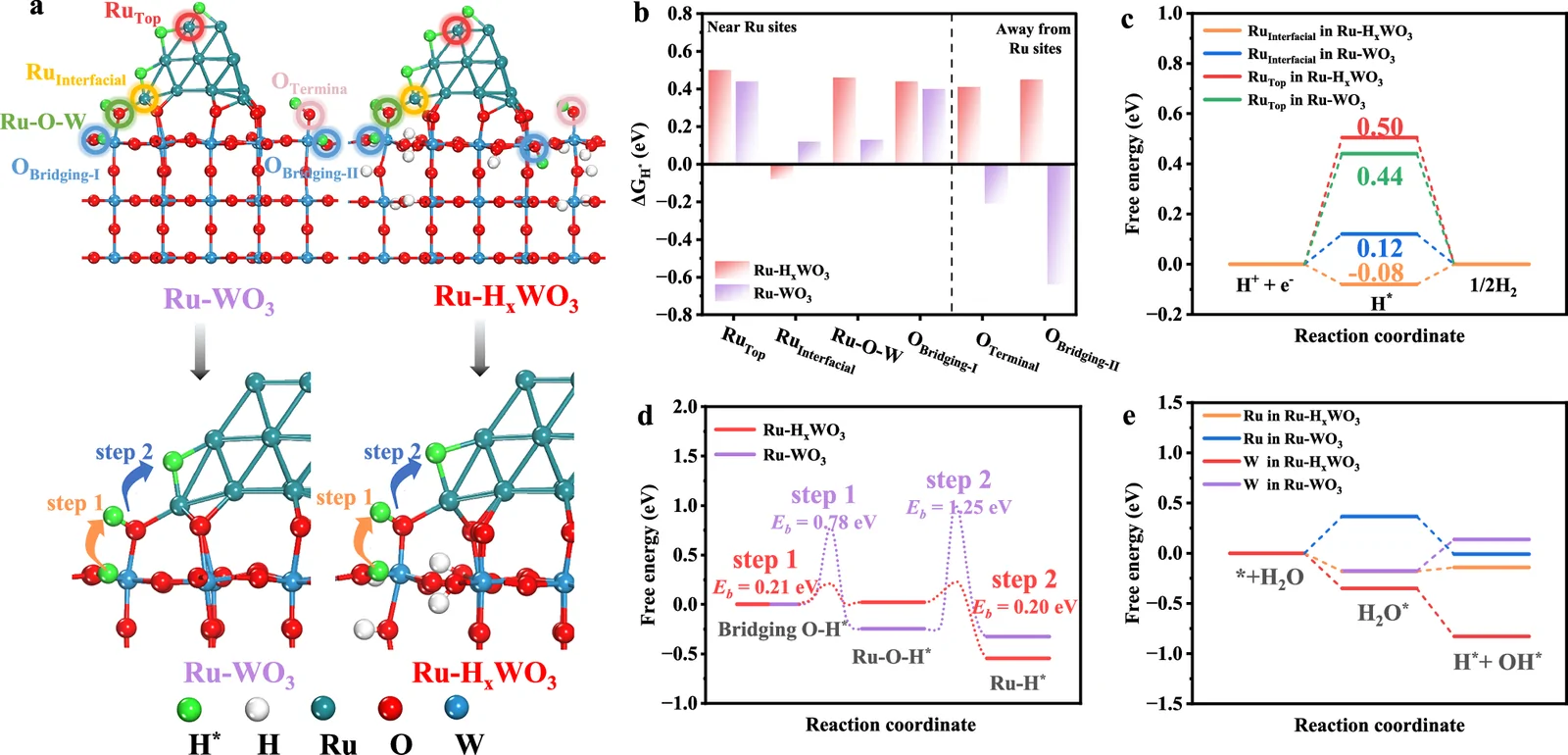
DFT calculation of the dynamic migration and replenishment of lattice-H. **a,b** Schematic diagram of different sites for H adsorption and migration near the interface (**a**) and corresponding adsorption free energy (**b**) in Ru-WO_3_ and Ru-H_x_WO_3_. **c** Free energy profiles for HER on Ru sites in Ru-H_x_WO_3_. **d** Hydrogen migration energy barriers corresponding to step 1 and step 2 in (**a**). **e** Free energy profiles for water dissociation on Ru and W sites in Ru-WO_3_ and Ru-H_x_WO_3_.

To further understand the hydrogen migration, evolution, and replenishment during the HER process, DFT calculations were conducted based on the optimized models of WO_3_, H_x_WO_3_, Ru-WO_3_ and Ru-H_x_WO_3_, as shown in Fig. 5 (Supplementary Fig. 30). First, the free energies of H adsorption (Δ*G*_H*_) on all possible active sites are calculated (Fig. 5a–c). For WO_3_, both the terminal and bridging O sites on its surface display highly negative H adsorption free energies (Supplementary Fig. 31), which results in the easy H insertion but poor HER performance. In the case of Ru-WO_3_, the Δ*G*_H*_ values of all Ru sites are positive (Fig. 5b, c). The Δ*G*_H*_ for the terminal O atoms connecting with Ru (i.e., Ru-O) and the bridging O atoms near Ru (O_Bridging-Ⅰ_) turn positive, while those for both the terminal O and bridging O sites away from Ru (i.e., O_Terminal_ and O_Bridging-Ⅱ_) remain negative (Fig. 5b). Consequently, the H atom preferentially adsorbs on the WO_3_ support rather than on Ru sites. This result aligns with the inferior lattice-H migration ability of the WO_3_ support and the relatively poor HER performance of Ru-WO_3_ (Figs. 4b and 3d). On the contrary, for Ru-H_x_WO_3_, which contains abundant lattice-H atoms, all O sites exhibit positive Δ*G*_H*_, which is conducive to the migration of lattice-H atoms to the Ru sites (Fig. 5b). Moreover, the Δ*G*_H*_ for the Ru_Interfacial_ in H_x_WO_3_ is -0.08 eV, very close to the ideal value for HER. In contrast, the Δ*G*_H*_ for the possible Ru and W active sites remain highly positive, showing the inferior HER activity of these sites (Fig. 5b,c, Supplementary Fig. 32)^33^. As a result, a feasible pathway for the lattice-H migration is established at the interface of Ru-H_x_WO_3_, which involves the lattice-H migration from the O_Bridging-Ⅰ_ to the terminal O, and further to the interfacial Ru sites (i.e., step 1 and step 2 in Fig. 5a). Through Climbing Image Nudged Elastic Band (CI-NEB) calculations, it was determined that the kinetic migration energy barriers in Ru-H_x_WO_3_ are quite small, only 0.21 eV in step 1 and 0.20 eV in step 2, which are significantly lower than the corresponding 0.78 and 1.25 eV in Ru-WO_3_, respectively (Fig. 5d). This finding indicates that, in Ru-H_x_WO_3_, it is easy to realize the lattice-H migration to the highly active HER sites, i.e., the interfacial Ru sites with a near-zero Δ*G*_H*_ (-0.08 eV), which leads to competitive HER performance (Fig. 3)^33^.

The lattice-H atoms consumed at the interfacial Ru can be readily replenished by the rapid hydrogen migration from the WO-H sites away from the Ru sites via H hopping between O sites with low energy barriers (e.g., <0.6 eV) (Fig. 4b, and Supplementary Fig. 22)^34^. The lattice-H atoms can be sourced from the electrolytes. In acidic electrolyte, due to the strong affinity between H^+^ ions and the O sites of H_x_WO_3_, H^+^ ions can adsorb onto these sites to form lattice-H atoms (Supplementary Fig. 31). In alkaline or neutral electrolytes, the lattice-H is replenished through water dissociation, which is, however, often hindered by sluggish kinetics. To understand this phenomenon, we calculated the water dissociation process on each metal site in Ru-WO_3_ and Ru-H_x_WO_3_ (Supplementary Fig. 31). The results show that for Ru-WO_3_, H_2_O molecules tend to adsorb on the W sites rather than the Ru sites. In contrast, for Ru-H_x_WO_3_, H_2_O molecules can spontaneously adsorb on both Ru and W sites, with the W sites having a more negative adsorption energy, which is confirmed by the contact angle (Supplementary Fig. 33). Unlike the W sites in Ru-WO_3_ and the Ru sites in Ru-H_x_WO_3_, the W sites in Ru-H_x_WO_3_ can spontaneously dissociate the adsorbed water, as evidenced by its downhill trend of the free energy profile (Fig. 5e). Thus, the lattice-H in Ru-H_x_WO_3_ can be easily replenished.

The preceding experimental results, supported by theoretical studies, validate a lattice-H cycling mechanism for the HER of Ru-H_x_WO_3_ NN. The H_x_WO_3_ NN support with abundant lattice-H obtained by thermal hydrogenation of WO_3_ NN can serve as a “H reservoir” and continuously supply H species to the highly-active HER sites of the interfacial Ru sites (Δ*G*_H*_ = -0.08 eV), leading to the competitive HER performance via the Tafel process (Supplementary Fig. 17). The consumed lattice-H in H_x_WO_3_ support is spontaneously replenished in all-pH electrolytes through H adsorption (acidic) or water dissociation (alkaline/neutral). A rapid lattice-H migration pathway is established from non-proton-blocking support (H_x_WO_3_) to proton-blocking metal catalysts (Ru) through H hopping between O sites. Such a process reduces dependence on the pH of the used electrolytes. This strategy should also be applicable to some other combination of non-proton-blocking support with proton-blocking metal catalysts. In fact, by replacing Ru with Ir or Pt, the corresponding Ir-H_x_WO_3_ NN and Pt-H_x_WO_3_ NN did exhibit much better pH-universal HER performances than Ir-WO_3_ NN and Pt-WO_3_ NN, respectively (Supplementary Fig. 34).

## Discussion

This study addresses the critical challenge of designing pH-universal HER electrocatalysts capable of operating efficiently at industrial current densities. By a lattice-hydrogen cycling mechanism, we decouple H^*^ availability from electrolyte pH, enabling robust HER performance across acidic, neutral, and alkaline environments. The integration of proton-blocking Ru nanoparticles with thermally-hydrogenated H_x_WO_3_ nanoneedles creates a dynamic hydrogen reservoir, where pre-inserted lattice hydrogen in H_x_WO_3_ migrates swiftly to Ru active sites via low-energy pathways (0.20–0.21 eV). Meanwhile, the consumed hydrogen is replenished through proton adsorption (acidic) or water dissociation (alkaline/neutral), ensuring sustained catalytic activity. In situ Raman spectroscopy, isotopic labeling, and DFT calculations confirm the lattice-hydrogen migration and replenishment processes. As a result, the Ru-H_x_WO_3_ catalyst achieves a competitive pH-universal HER performance with low overpotentials of 125 mV (0.5 M H₂SO₄), 219 mV (1 M PBS), and 142 mV (1 M KOH) at 1 A cm^-2^, alongside 500-hour stability. The broader utility of the design strategy for pH-universal catalysts is also demonstrated by the applicability to Ir and Pt-based systems. Further attention should be paid to the validation of the mechanism’s efficacy in other hydrogen-involving reactions, such as CO_2_RR, NRR, and other renewable energy-driven processes. Additionally, real-world conditions such as fluctuating renewable energy inputs and seawater electrolysis are expected to be explored.

In a short, this work establishes a groundbreaking strategy for pH-robust electrocatalysts, bridging fundamental insights with industrial relevance. By overcoming pH-dependent kinetic limitations, the lattice-hydrogen cycling mechanism paves the way for sustainable energy conversion systems, aligning with global decarbonization goals.

## Methods

### Materials

Sodium tungstate dihydrate (Na_2_WO_4_·2H_2_O, AR), ammonium sulfate ((NH_4_)_2_SO_4_, AR), sodium sulfate (Na_2_SO_4_, AR), and deuteroxide (D_2_O, AR) were purchased from Shanghai Macklin Biochemical Co., Ltd. Potassium phosphate monobasic (KH_2_PO_4_, AR) and potassium hydrogen phosphate (K_2_HPO_4_, AR) were obtained from Shanghai Aladdin Bio-Chem Technology Co., Ltd. Potassium hydroxide (KOH, AR), hydrochloric acid (HCl, AR, 36.0–38.0%), sulfuric acid (H_2_SO_4_, AR, 98%) oxalic acid dihydrate (H_2_C_2_O_4_·2H_2_O, AR), sodium borohydride (NaBH_4_, AR), acetone (C_3_H_6_O, AR) and ethanol (C_2_H_5_OH, AR) were purchased from Sinopharm Chemical Reagent Co., Ltd. Ruthenium (III) chloride anhydrous (RuCl_3_) were purchased from Tianjin Xiensi Biochemical Technology Co., Ltd. Ru/C (5 wt%), Pt/C (20 wt%), iridium oxide (IrO_2_, 99.9%) and Nafion 117 solution (5 wt%) were purchased from Sigma-Aldrich. Ir/C (5 wt%) was purchased from Meryer Chemical Technology Co.,Ltd. Cu foam (CF, thickness: 1.6 mm) was purchased from Shanghai Tankii Alloy Material Co., Ltd.

### Synthesis of WO_3_ NN

A piece of CF (2 × 4 cm) was etched in HCl solution (1 M) for ~10 min to eliminate the surface oxide layer. Then, it was washed successively with acetone, ultra-pure water and ethanol under ultrasonic condition. WO_3_ nanoneedle (NN) arrays were grown on CF by a simple hydrothermal process. Na_2_WO_4_·2H_2_O (3 mmol) and H_2_C_2_O_4_ (8 mmol) were dissolved in 30 mL of ultra-pure water with 250 μL HCl (36.0-38.0%). Subsequently, (NH_4_)_2_SO_4_ (6 mmol) and Na_2_SO_4_ (6 mmol) were added into the solution under continuous magnetic stirring for 20 min. The resultant solution was transferred into a 50 mL autoclave and the pre-treated CF was immersed in the solution. The autoclave was heated to 180 °C and maintained at this temperature for 16 h. The obtained WO_3_ NN arrays on CF were washed with deionized water and ethanol for several times, and dried at 80 °C for 10 h.

### Synthesis of Ru-H_x_WO_3_ NN

RuCl_3_ (50.0 mg) was dissolved in 20.0 mL of 0.1 M HCl solution through magnetic stirring combined with ultrasonication to prepare the RuCl_3_ solution (2.5 mg mL^-1^). Subsequently, WO_3_ NN on CF was immersed in the RuCl_3_ solution for 1 h. After that, it was washed with deionized water and ethanol for several times, and dried at 80 °C for 2 h. The product was placed into a tubular furnace and heated to 400 °C for 2 h under H_2_ atmosphere to obtain Ru-H_x_WO_3_ NN. H_x_WO_3_ NN on CF was obtained by following the above procedure while omitting immersing in the RuCl_3_ solution. The mass loading of Ru-H_x_WO_3_ on CF was controlled to be ~8 mg cm^-2^. The Ir-H_x_WO_3_ and Pt-H_x_WO_3_ NN were prepared by a similar process, with the exception that RuCl_3_ was replaced by IrCl_3_ and H_2_PtCl_6_ solution, respectively.

### Synthesis of Ru-WO_3_ NN

NaBH_4_ (160.0 mg) was dissolved in 20.0 mL of 1 M NaOH solution to prepare the NaBH_4_ solution (8.0 mg mL^-1^). Subsequently, WO_3_ NN on CF was immersed in the RuCl_3_ solution for 1 h. After that, it was washed with deionized water and ethanol for several times, and dried at 80 °C for 2 h. The resultant product was immersed in the NaBH_4_ solution to reduce Ru, named as Ru-WO_3_ NN. The mass loading of Ru-WO_3_ on CF was controlled to be ~8 mg cm^-2^. The Ir-WO_3_ and Pt-WO_3_ NN were prepared by a similar process, with the exception that RuCl_3_ was replaced by IrCl_3_ and H_2_PtCl_6_ solution, respectively.

### Preparation of Pt/C and Ru/C electrodes

To prepare the Pt/C or Ru/C electrodes, 32 mg commercial Pt/C or Ru/C, 100 μL Nafion and 900 μL ethanol were ultrasonicated for 60 min to obtain a homogeneous ink. Then, the ink was coated onto a piece of cleaned CF (2 × 2 cm), followed by drying at 80 °C for 10 h. The mass loading of Pt/C or Ru/C on CF was controlled to be ~8 mg cm^-2^.

### Preparation of NiFe-LDH electrodes

The NiFe-layered double hydroxide (NiFe-LDH) was synthesized by a three-electrode system, with the CF (3 × 2 cm), a graphite rod and a saturated calomel electrode (SCE) as the working, counter and reference electrodes, respectively, and 100 mL of solution containing 0.015 mol Ni(NO_3_)_2_·6H_2_O and 0.015 mol FeSO_4_·7H_2_O as electrolyte. The electrodeposition was carried out under the N_2_ atmosphere at a constant potential of -1.0 V vs. SCE for 600 s.

### Materials characterization

X-ray diffraction (XRD) patterns were recorded on a Bruker D2 Advance A25 using a Cu K_α_ radiation of 1.5406 Å at 30 kV and 10 mA. The morphology and structure were characterized by scanning electron microscope (SEM, Hitachi, S-8100), high-resolution transmission electron microscope (HRTEM, JEM-2100) and high-angle annular dark-field scanning transmission electron microscopy (HAADF-STEM, Thermo Fisher Scientific, FEI Titan Themis 60−300) equipped with energy dispersive X-ray spectrometer (EDS, SUPER X). The Raman spectra were recorded by a confocal Raman spectrometer (Horiba JY Raman) with a laser of 473 nm. ^1^H solid-state nuclear magnetic resonance (NMR) measurements were performed at room temperature on a Bruker 400 MHz NMR spectrometer. Mass spectrometry was recorded on NETZSCH STA 449F3-QMS 403 C Aëolos. X-ray absorption fine structure (XAFS) measurements were carried out on the BL11B beamline of the Shanghai Synchrotron Radiation Facility (SSRF). The obtained XAFS data were analyzed and fitted by using Athena and Artemis software. The surface chemical states and the element composition were analyzed by X-ray photoelectron spectroscopy (XPS, ULVAC-PHI INC, PHI 5000 VersaProbe) with an Al X-ray source, and XPS spectra were calibrated with the C 1 *s* peak at 284.8 eV.

### Thermogravimetric analysis

The WO_3_ NN powder ( ~ 50 mg) with a little Cu scraped from CF was placed in a crucible and heated to 400 °C in H_2_/Ar (10%H_2_/90%Ar) or pure Ar atmosphere with a heating rate of 5 °C min^-1^ for 2 h. The thermogravimetric analysis (TGA) and derivative thermogravimetry (DTG) curves were recorded by NETZSCH STA 449F3 instrument.

### Electrochemical measurements

The electrochemical measurements were conducted on Biologic VMP3 electrochemical workstation in a three-electrode system, with a graphite rod with a diameter of 8 mm as counter electrode and the catalysts on Cu foam (1 × 1 cm) as the working electrodes. The Hg/HgO, SCE, and Hg/Hg_2_SO_4_ electrode were chosen as reference electrodes in 1 M KOH, 1 M phosphate buffered solution (PBS), and 0.5 M H_2_SO_4_, respectively. The 1 M PBS (pH=7.0) was prepared by mixing 1 M K_2_HPO_4_ with 1 M KH_2_PO_4_ in a volume ratio of 2:1. The working electrodes were initially activated through cyclic voltammetry (CV). The CV was performed at a scan rate of 50 mV·s^−1^ for 30 cycles within a potential range from -0.1 to 0.1 V versus reversible hydrogen electrode (vs. RHE). The hydrogen evolution reaction (HER) polarization curves were measured in N_2_-saturated solution via linear sweep voltammetry (LSV) at a sweep rate of 2 mV s^−1^. To measure the double-layer capacitances (C_dl_), CV was carried out at different scan rates from 10 to 100 mV s^−1^. Subsequently, the electrochemical active surface area (ECSA) could be calculated by the formula ECSA = C_dl_/C_s_, where C_s_ is the specific capacitance with an average value of 0.040 mF cm^-2^. Electrochemical impedance spectroscopy (EIS) was conducted within a frequency range from 0.1 Hz to 100 kHz with an amplitude of 5 mV. The long-term durability was evaluated through chronoamperometry (CP) measurements at a constant current density and room temperature, and the electrolyte was replaced every 48 h. All the measured potentials versus Hg/HgO electrode, SCE, and Hg/Hg_2_SO_4_ electrode were converted to potentials versus RHE according to the equation of *E*_RHE_ = *E*_Hg/HgO_ + 0.059×pH + 0.098, *E*_RHE_ = *E*_SCE_ + 0.059×pH + 0.241, *E*_RHE_ = *E*_Hg/Hg2SO4_ + 0.059×pH + 0.653, respectively. All the polarization and CP curves in this study were corrected for *iR*_s_ compensation, and the *iR* formula is *E*_*iR*-correction_*=E*_test_ *- iR*_s_, where *R*_s_ was measured by EIS tests at 0 V vs. reference electrodes in different electrolytes. To get the activity of the catalyst per unit mass at the overpotential of 100 mV, the mass activities are calculated by the formula mass activities = j_100mV_/m(Matal), where j represents the current density at the overpotential of 100 mV and m represents the mass of the precious metal.

### Electrochemical Measurements in AEMWE

The membrane electrode assembly (MEA) for the anion exchange membrane water electrolyzer (AEMWE) consisted of a Ru-H_x_WO_3_ NN cathode, a NiFe-LDH anode and a commercial AEM membrane (FAA-3-PK-130). The active area of electrode was 2 cm×2 cm. The AEMWE device was operated at 80 °C with 1 M KOH or 1 M KOH + 0.5 M NaCl as the electrolytes under a flow rate of 20 mL min^–1^. Before the test, AEMWE was activated for 1 h at a current of 0.5 A. The polarization curves were recorded by changing the current density from 0.01 to 1 A cm^-2^. The overall AEMWE device showed the resistance (R) of ~0.25 Ω in 1 M KOH and ~0.2 Ω in 1 M KOH + 0.5 M NaCl. The polarization curves were corrected by *iR* compensation.

### Measurement of pH on the catalyst surface

The pH values on the catalyst surface were measured via the rotating ring-disk electrode (RRDE) technique, which was carried out on a CHI 760E electrochemical workstation (Shanghai CHI Instruments Company)^26^. The potential of Pt ring electrode (RE, 0.1866 cm^2^) is sensitive to pH and can be used to monitor the variations in the pH on the surface of disk electrode (DE, 0.2475 cm^2^) surface. The RRDE and graphite rod were used as working electrode and counter electrode, respectively. The Hg/HgO, SCE, and Hg/Hg_2_SO_4_ electrodes were chosen as reference electrodes for H_2_SO_4_, PBS and KOH solutions, respectively. Then, the pH dependence of open circuit potential (OCP) was measured with Pt RE in H_2_-saturated H_2_SO_4_, PBS and KOH solutions. The pH of H_2_SO_4_ and KOH solution was adjusted by adding 0.5 M H_2_SO_4_ or 1 M KOH to 0.5 M K_2_SO_4_. The pH of PBS solution was adjusted by adding 1 M H_3_PO_4_ or 1.5 M KOH to 1 M PBS. The OCP of the Pt electrode represents the equilibrium potential of the reaction 2H^+^ + 2e^−^ → H_2_, which varies with pH according to the Nernst equation:

1 $${E}_{R{{{\rm{ocp}}}}}\left(V{{{\rm{vs}}}}.{{{\rm{reference\; electrode}}}}\right)=\frac{-2.303{RT}}{F}{{{\rm{pH}}}}$$

The fugacity of H_2_ is assumed to be 1 and *R*, *T*, and *F* are the gas constant, the absolute temperature, and the Faraday constant, respectively.

To measure the pH on the electrode surface, the catalyst was loaded onto the disk electrode. The catalyst ink was prepared by ultrasonically dispersing catalyst powder (25 mg) scraped from CF in a mixed solution of 5 wt% Nafion solution (80 μL), ethanol (200 μL) and ultra-pure water (720 μL). Then, 10 μL of catalyst ink was dropped onto the disk electrode with catalyst loading of 1 mg cm^−2^. A constant potential method was applied to the disk electrode (*E* = 0.1, 0, -0.1, -0.2, -0.3, -0.4, -0.5, -0.6, -0.7 V vs. RHE) for 300 s, and the OCP was simultaneously measured on the Pt ring electrode. The pH value of the catalyst-loaded DE can be deducted from the pH value of the Pt RE by the following equation:

2 $${c}_{{R,{{{\rm{H}}}}}^{+}}-{c}_{R,{{{{\rm{OH}}}}}^{-}}={N}_{D}\left({c}_{{D,{{{\rm{H}}}}}^{+}}-{c}_{{D,{{{\rm{OH}}}}}^{-}}\right)+(1-{N}_{D})({c}_{{\infty,{{{\rm{H}}}}}^{+}}-{c}_{{\infty,{{{\rm{OH}}}}}^{-}})$$

where ${c}_{{R,{{{\rm{H}}}}}^{+}}$ and ${c}_{{D,{{{\rm{H}}}}}^{+}}$ are the concentrations of H^+^ on the RE and DE, respectively; ${c}_{R,{{{{\rm{OH}}}}}^{-}}$ and ${c}_{{D,{{{\rm{OH}}}}}^{-}}$ are the concentrations of OH^-^ on the RE and DE, respectively; ${c}_{{\infty,{{{\rm{H}}}}}^{+}}$ and ${c}_{{\infty,{{{\rm{OH}}}}}^{-}}$ are the concentrations of H^+^ and OH^-^ in the bulk electrolyte, respectively. N_*D*_ = 0.37 is the collection efficiency of the RE.

### In situ Raman spectroscopy measurements

In situ Raman spectra were recorded by a confocal Raman spectrometer (Horiba JY Raman, with a laser of 473 nm from an argon-ion laser) under specific potentials controlled by CHI 760E electrochemical workstation. A Pt plate and a SCE electrode were employed as the counter and reference electrode, respectively. The catalysts on CF were directly used as the working electrode. CP measurements were carried out within a specific potential range with the interval of 50 mV in different electrolytes (0.5 M H_2_SO_4_, 1 M PBS, and 1 M KOH). Raman tests were initiated once the CP test had exceeded 30 s, and the acquisition time was set to 45 s. For time-resolved in situ Raman spectroscopy, the 1 M PBS and 1 M KOH dissolve in D_2_O solvent and CP measurements were conducted at constant potential of -0.1 V vs. RHE.

### DFT calculation

Density functional theory (DFT) calculations with the plane-wave basis set were performed using the Vienna Ab Initio Simulation Package (VASP)^35^. The exchange-correlation functional was the Revised Perdew-Burke-Emzerhof (RPBE)^36^ of parametrization of the generalized gradient approximation. The electron-ion interactions were described by the projector augmented wave (PAW). The van der Waals interactions were corrected by the DFT-D3 method with Becke-Johnson damping function^37^. A plane-wave basis was set with the cutoff energy of 450 eV. A Gamma-centered 3 × 3 × 1 k-point mesh for Brillouin zone integration was used for static calculations and a 1 × 1 × 1 mesh for structure optimizations. A 2 × 2 × 1 supercell was built from (002) surface of WO_3_ with a 23.5 Å vacuum along the z-axis and the lattice parameter as 14.8 × 14.8 × 35.0 Å. H_x_WO_3_ and Ru-H_x_WO_3_ models were constructed base on this slab. VASPKIT was used to assist various computational tasks^38^. Geometry optimization was performed until the convergence criteria were satisfied, with force converging to less than 0.04 eV Å^−1^ and energy difference between ionic steps of less than 10^−6^ eV. VASPsol package was used for counting the implicit solvent effects. The XAFS calculations were carried out with FDMNES code^39^.Transition state searches were conducted using the climbing image nudged elastic band (CI-NEB) method^40^, as implemented in the VTST (VASP Transition State Tools) package^41^. The Gibbs free energy of each reaction state was calculated by:

3 $${{{\rm{G}}}}={E}_{{{{\rm{DFT}}}}}+{E}_{{{{\rm{ZPE}}}}}-{TS}$$

The adsorption Gibbs free energy was calculated by:

4 $${{\Delta }G}_{{{{\rm{adsorption}}}}}={G}_{{{{\rm{slab}}}}*{{{\rm{H}}}}}-{G}_{{{{\rm{slab}}}}}-{1/2G}_{{{{\rm{H}}}}2}+{|e|}{U}_{{{{\rm{SHE}}}}}+0.0592\times {{{\rm{pH}}}}$$

## Supplementary information

Supplementary Information

Description of Additional Supplementary Files

Supplementary Data 1

Transparent Peer Review file

## Source data

Source Data

Source Data for Supplementary Information

## Data Availability

All data supporting the findings of this study can be found in the main text and the Supplementary Materials, or are available from the corresponding authors upon request. The source data generated in this study are provided in the Source Data file and Supplementary Data 1. Source data are provided with this paper.
