# Supplementary material for: Lattice-hydrogen cycling mechanism enables pH-universal hydrogen evolution at ampere-level current densities
Source: Nat Commun. 2025 Dec 3;16:10863. doi: 10.1038/s41467-025-65909-3 (PMC12675675; doi:10.1038/s41467-025-65909-3)
Supplement: Supplementary file 1 — Supplementary Information [file 41467_2025_65909_MOESM1_ESM.pdf]

Supplementary Materials for

Lattice-Hydrogen Cycling Mechanism Enables pH-Universal Hydrogen Evolution at Ampere-Level Current Densities

Yan Zhang†, Biao Feng†, Jingyi Tian, Shiqi Zhou, Changkai Zhou, Yiqun Chen, Xiaoli Xia, Xizhang Wang, Lijun Yang, Luming Peng, Qiang Wu*, Hongwen Huang*, and Zheng Hu*

*Corresponding author. Email: wqchem@nju.edu.cn; huanghw@nju.edu.cn; zhenghu@nju.edu.cn

†These authors contributed equally to this work.

This file includes:

Supplementary Figs. 1 to 34
Supplementary Tables S1 to S7

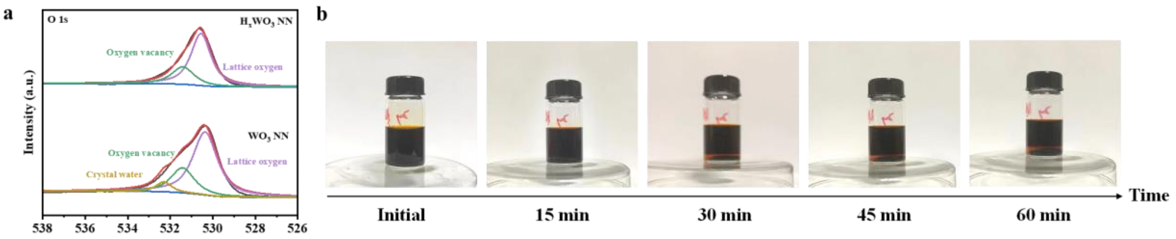

Supplementary Fig. 1. a O 1*s* XPS spectra of WO_3 NN and H_xWO_3 NN. **b** Photos of $RuCl_3$ impregnation process at different time

The O1*s* spectra reveal the presence of abundant oxygen vacancies in WO_3 NN and H_xWO_3 NN (Supplementary Fig. 1a). These oxygen vacancies play a crucial role in facilitating the immobilization of Ru^{3+} species through interactions [8]. Over time, the color of the $RuCl_3$ solution gradually fades, indicating the progressive deposition of Ru species onto H_xWO_3 NN (Supplementary Fig. 1b).

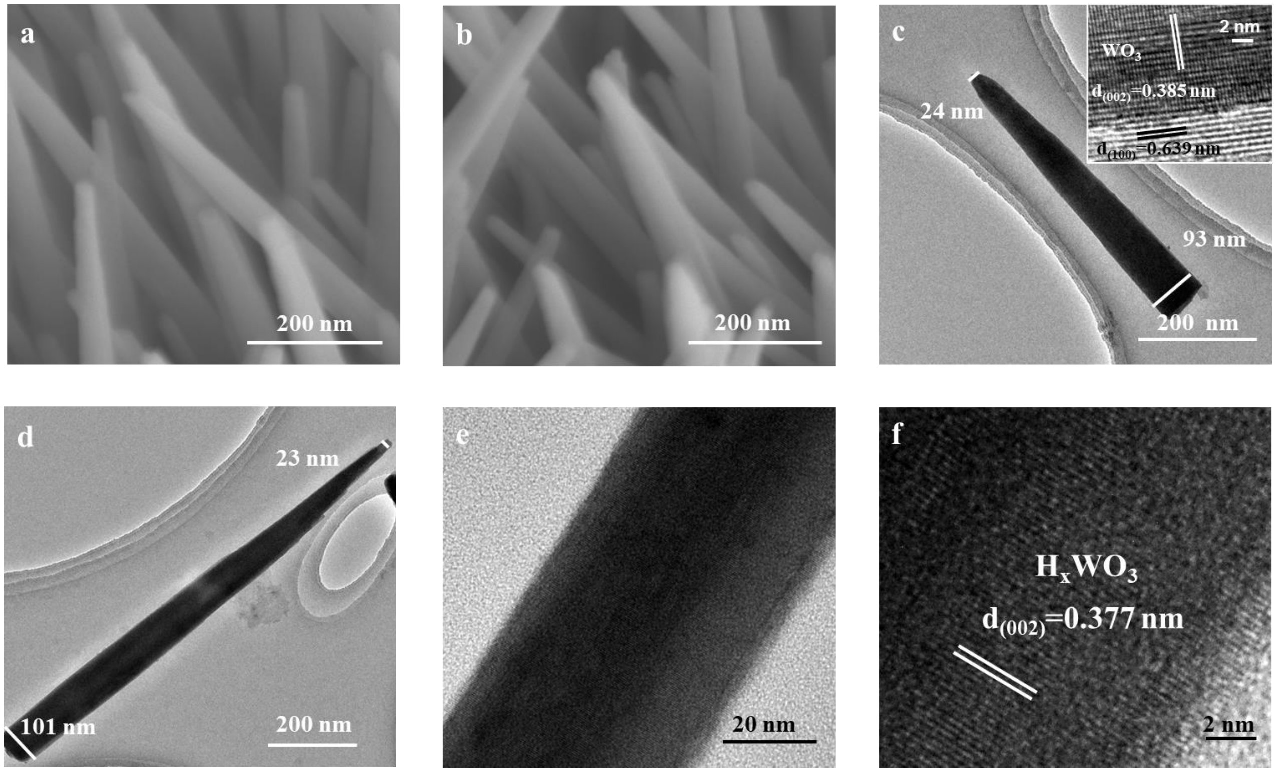

Supplementary Fig. 2. Morphology of WO_3 NN and H_xWO_3 NN. a,b SEM images of WO_3 NN (**a**) and H_xWO_3 NN (**b**). **c-e** TEM images of WO_3 NN (**c**) and H_xWO_3 NN (**d,e**). Inset in (**c**) is the corresponding HRTEM image. **f** HRTEM image of H_xWO_3 NN.

The WO_3 NN shows the lattice distances of 0.385 nm and 0.639 nm, corresponding to the (002) and (100) planes in the radial and axial direction, respectively (Supplementary Fig. 2c). The H_xWO_3 NN retains the nanoneedle morphology and size similar to those of WO_3 NN (Supplementary Fig. 2a-d). The H_xWO_3 NN features a smooth surface, and it is noted that the interplanar distance for (002) plane is compressed to 0.377 nm (Supplementary Fig. 2e,f).

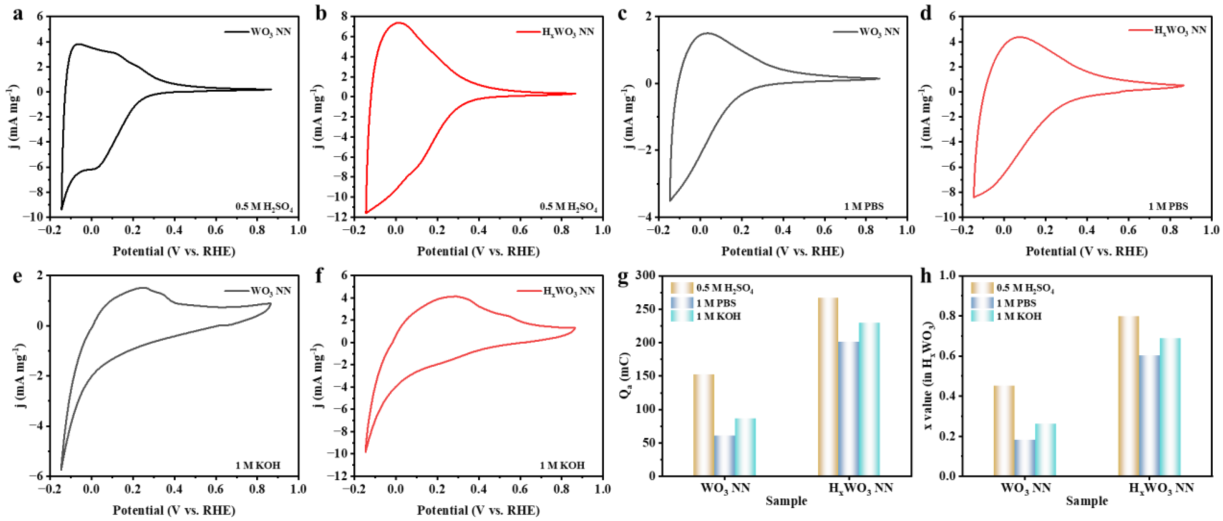

Supplementary Fig. 3. CV curves, deduced Q_a and *x* value in 0.5 M H_2SO_4, 1 M PBS and 1 M KOH. a-f CV curves. **g,h** deduced Q_a and x value.

The CV tests were used to quantify the amount of reversible hydrogen insertion in WO_3 NN and H_xWO_3 NN[9]. The WO_3 NN and H_xWO_3 NN powders were stripped from the Cu foam by ultrasonication, and then coated on carbon paper as working electrode with a mass loading of 1 mg cm^{-2}. The CV scan rate is 10 mV s^{-1}. The integral of the positive current with respect to time for one CV cycle is taken to calculate the Q_a, which was converted to the number of hydron atom per tungsten atom by the following equations:

per tungsten atom by the following equations:

$$Q_a = \int j\mathrm{d}t \quad (1)$$

$$n_H = \frac{Q_a \times N_e}{N} \quad (2)$$

$$x = \frac{n_H}{n_{WO_3}} \quad (3)$$

where j is the positive current, n_H is the amount of substance for H insertion, N_e is the number of electrons per Coulomb; N is the Avogadro constant; n_{WO_3} is the amount of substance for WO_3.

It was found that after the H insertion via electrochemical method, the x value of WO_3 NN was 0.45 in 0.5 M H_2SO_4, similar with the above report; and the x value of H_xWO_3 NN was 0.80 in 0.5 M H_2SO_4 which was obviously higher than that of WO_3 NN. For H_xWO_3 NN, the measured x value was the amount of H that can achieve reversible hydrogen insertion.

In addition, the x values of WO_3 NN were much lower when tested in neutral (x=0.18) and alkaline (x=0.26) electrolytes due to the increased difficulty in obtaining H species, indicating a poor hydrogen supply for WO_3 NN during HER. However, the x values of H_xWO_3 NN were 0.6 in neutral electrolyte and 0.68 in alkaline electrolyte, thanks to its high initial concentration and faster replenishment of lattice hydrogen, which is contributed to achieving similar HER performance in different electrolyte.

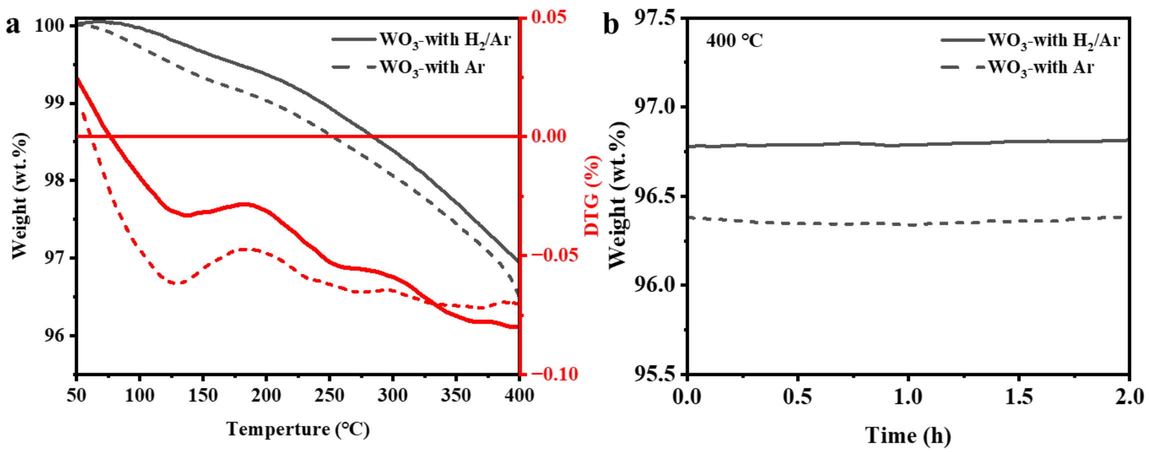

Supplementary Fig. 4. TGA results under H_2/Ar or Ar atmosphere. a TGA (black) and DTG (red) curves of WO_3 NN. **b** TGA curves of WO_3 NN at 400 °C.

The TGA curves of WO_3 NN were recorded under 10%H_2/90%Ar and pure Ar atmosphere, with the temperature ranging from room temperature to 400 °C, which exhibit a continuous downward trend due to the slight loss of crystal water. The DTG curves show that the weight-loss rate under the 10%H_2/90%Ar atmosphere is slower than that under the Ar atmosphere because of the H insertion into WO_3 (Supplementary Fig.4a).

Regarding the TGA curves of WO_3 NN at 400°C under the H_2/Ar or Ar atmosphere, the difference of weight loss is approximately 0.38-0.42 % (Supplementary Fig.4b), which can be used to calculate the value of x in H_xWO_3.

It is known that there is no change in the amount of substance (*n*) for WO_3 during TGA. Therefore, the value of *x* can be calculated using the following formula:

$$m_{\mathrm{H}} = m_{\mathrm{WO_3-H_2}} - m_{\mathrm{WO_3-Ar}} = \Delta\omega \times m_{\mathrm{initial}} \quad (4)$$

$$x \times n \times M_H = \Delta\omega \times n \times M_{\mathrm{WO_3}} \quad (5)$$

$$x = \frac{M_{\mathrm{wo_3}} \times \Delta\omega}{M_{\mathrm{H}}} \quad (6)$$

where *M* is the molecular weight, $\Delta\omega$ is the weight-loss difference, and *m* is the mass. Based on the TGA results, the x value in H_xWO_3 NN support is about 0.88-0.97.

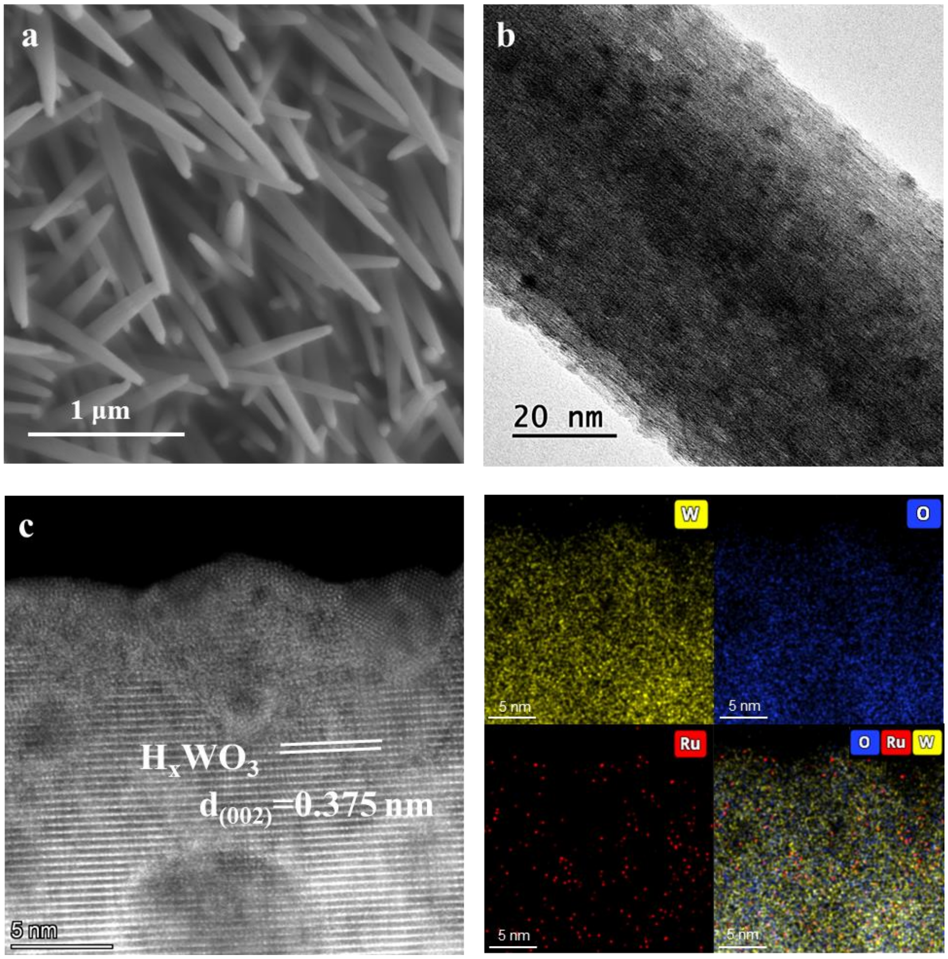

Supplementary Fig. 5. Morphology of Ru-H_xWO_3 NN. a,b SEM (**a**) and TEM (**b**) images. **c** HAADF-STEM image and corresponding EDS elemental mappings of W, Ru, O and their overlap.

Ru-H_xWO_3 NN retains the nanoneedle morphology and size similar to those of WO_3 NN (Supplementary Fig. 5a,b). The surface of Ru-H_xWO_3 NN is rough surface due to the presence of loaded nanoparticles (Supplementary Fig. 5b).

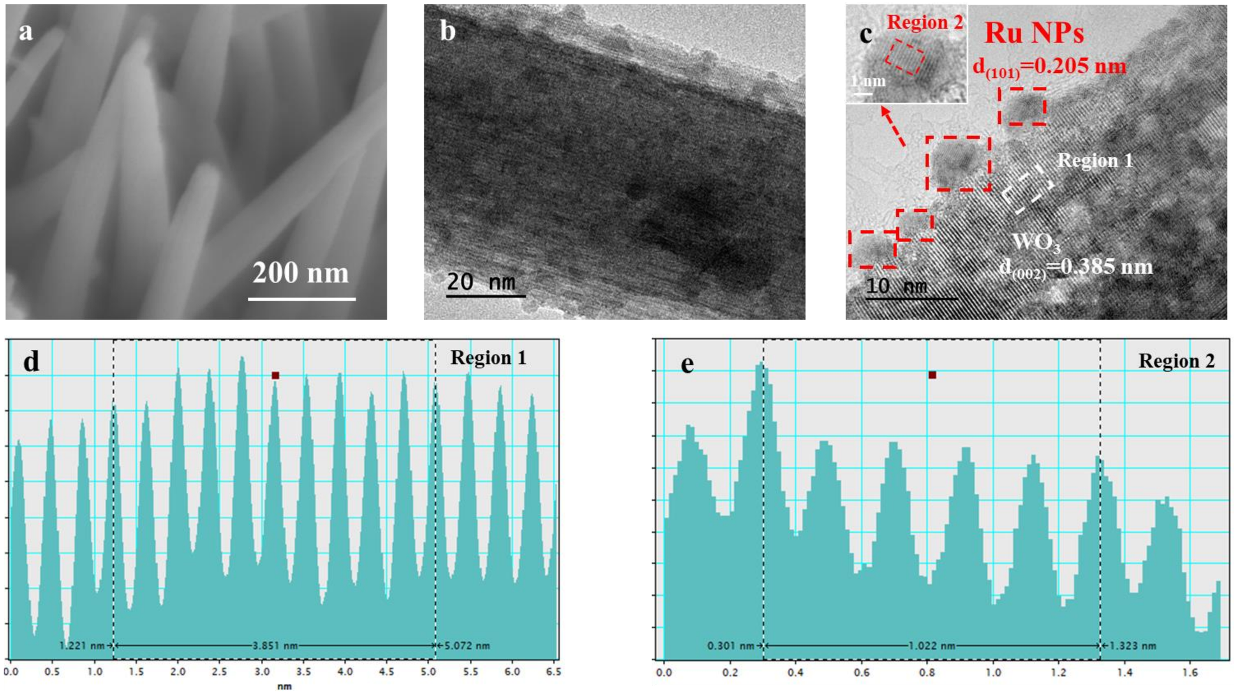

Supplementary Fig. 6. Morphology and crystal structure of Ru-WO_3 NN. **a-c** SEM (a), TEM (b) and HRTEM (c) image of Ru-WO_3 NN. Inset in (c) is the local enlargement. **d-e** The lattice spacing corresponding to the marked region 1 for WO_3 (d) and region 2 for Ru NPs (e).

Ru-WO_3 NN treated with the $NaBH_4$ solution shows a morphology and a loading density of Ru NPs similar to those of the Ru-H_xWO_3 NN, indicating negligible effect of $NaBH_4$ treatment on the morphology.

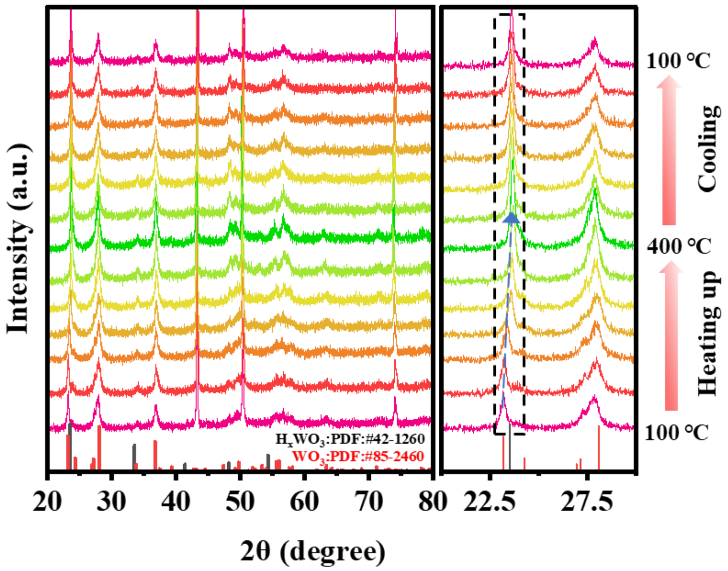

Supplementary Fig. 7. *In situ* XRD patterns for WO_3 sample thermal-hydrogenated at different temperatures under 10% H_2/Ar atmosphere. The local enlargements of the patterns in the range of 20°-30° are shown for clarity.

The WO_3 NN arrays on Cu foam was thermal-hydrogenated in 10% H_2/Ar atmosphere and the XRD patterns were *in situ* recorded on Smartlab SE with a temperature interval of 50 ℃ in the range of 100 ℃ to 400 ℃.

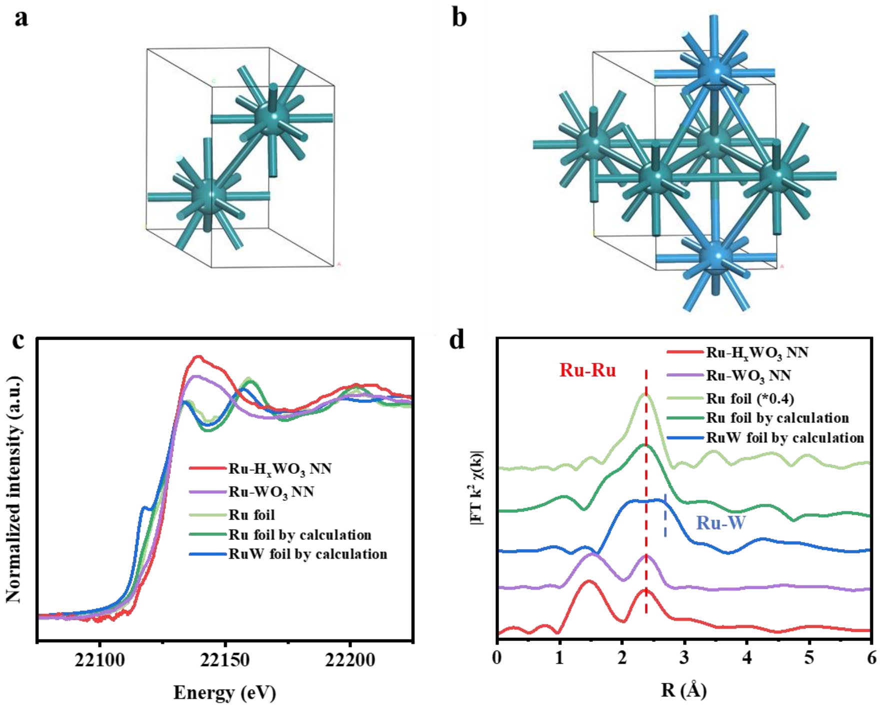

Supplementary Fig. 8. XAFS spectra by calculation. a,b Model of Ru foil (**a**) and RuW foil (**b**). **c** Ru K-edge XANES spectra. **d** Corresponding k^2-weighted R-space Fourier transformed EXAFS spectra for Ru.

To verify the validity of the calculation results, we first calculated the K-edge XANES spectrum and corresponding R-space EXAFS spectra for Ru of Ru foil, using FDMNES code[10]. It can be found that the calculated results are basically consistent with the experimental results. Then, the Ru K-edge XANES spectrum for RuW alloy foil and corresponding R-space EXAFS spectra for Ru in Ru and RuW foils were calculated. It is seen the Ru K-edge XANES for RuW alloy is obviously different from those of Ru foil and Ru-H_xWO_3 NN. In the EXAFS spectra, the Ru signal for Ru-W foil shows an obvious widening and position shift to ~2.7 Å, much different from the Ru foil and Ru-H_xWO_3 NN. Therefore, the Ru NPs bond to the H_xWO_3 and WO_3 NN through Ru-O ionic bonds, rather than Ru-W metallic bonds.

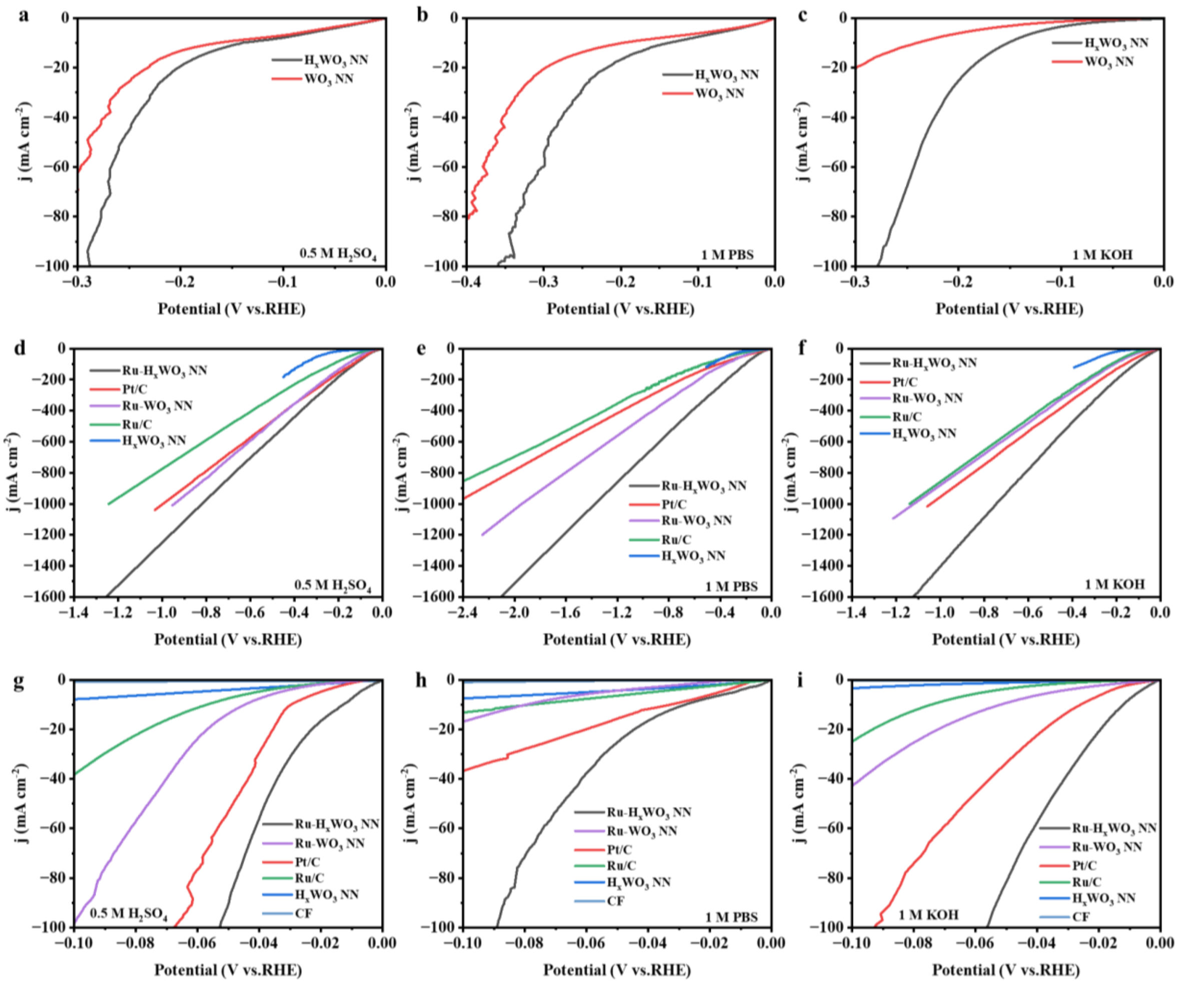

Supplementary Fig. 9. Polarization curves. a-c In 0.5 M H_2SO_4 (**a**), 1 M PBS (**b**) and 1 M KOH (**c**) of H_xWO_3 NN and WO_3 NN. **d-f** In 0.5 M H_2SO_4 (**a**), 1 M PBS (**b**) and 1 M KOH (**c**) without *iR* compensation. **g-i** In 0.5 M H_2SO_4 (**a**), 1 M PBS (**b**) and 1 M KOH (**c**) with low current range.

H_xWO_3 NN shows slightly superior HER performance compared to WO_3 NN in all-pH electrolytes.

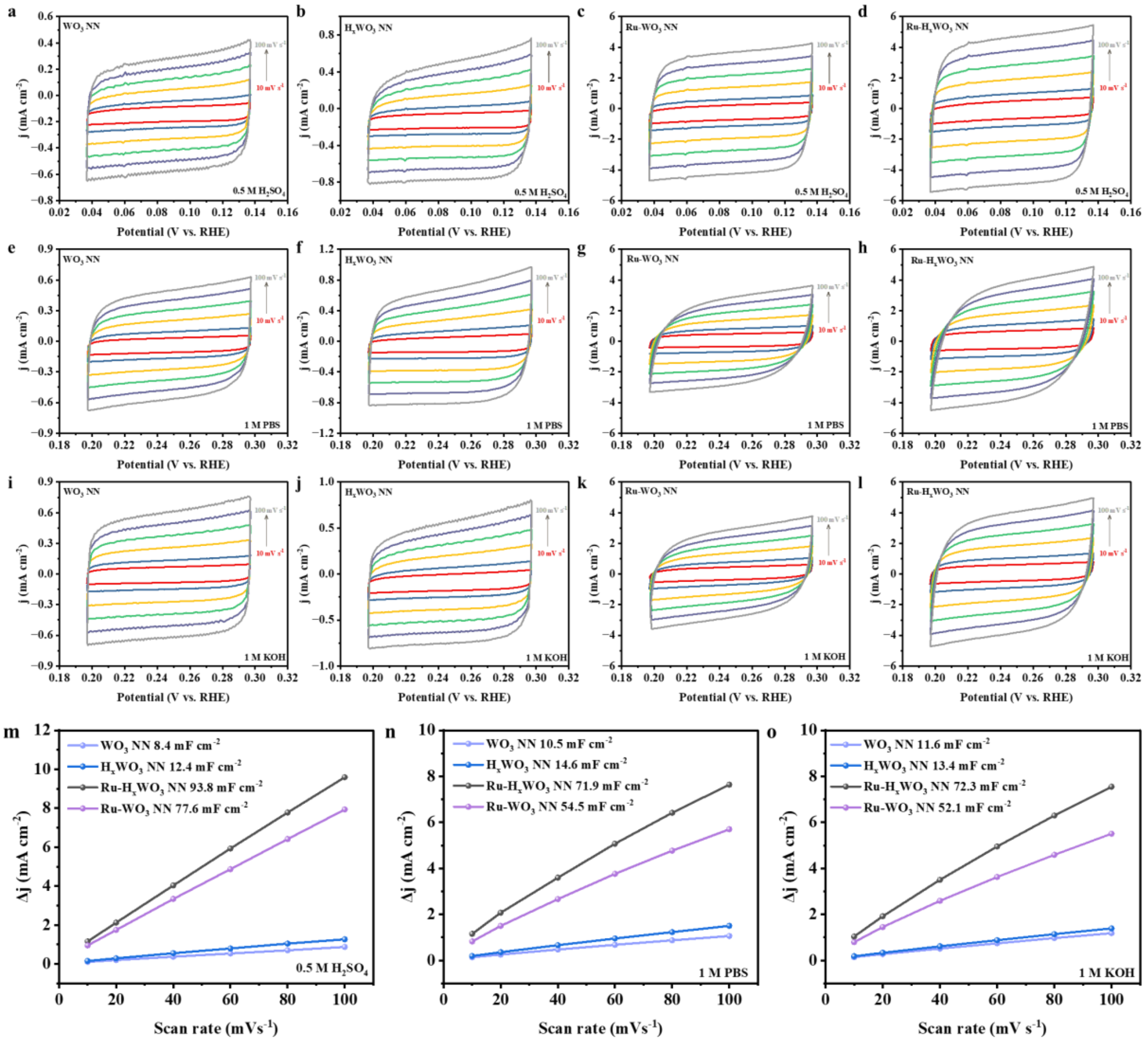

Supplementary Fig. 10. CV curves and deduced C_{dl} in 0.5 M H_2SO_4, 1 M PBS and 1 M KOH. **a-l** CV curves. **m-o** Deduced C_{dl} of different catalysts. The samples and electrolytes are marked in the figures.

To estimate C_{dl}, CV tests were carried out at the scan rates of 10, 20, 40, 60, 80, and 100 mV s^{-1} within the non-faradaic potential region. Specifically, the non-faradaic potential region was 0.197~0.297 V (vs. RHE) in KOH and PBS solutions while it was 0.038-0.138 V (vs. RHE) in H_2SO_4 solution. The lower potential range in H_2SO_4 solution was selected to avoid the Cu dissolution in acidic media at ~0.2 V (vs. RHE).

The capacitive current $\Delta j = (j_a - j_c)/2$ at the median potential of potential region was plotted versus the scan rate, where j_a and j_c represent the forward and reverse scanning current, respectively. Based on the CV curves, the C_{dl} values of Ru-H_xWO_3 NN were higher (93.8, 71.9, 72.3 mF cm^{-2} in 0.5 M H_2SO_4, 1 M PBS and 1 M KOH, respectively) than those of WO_3 NN, H_xWO_3 NN and Ru-WO_3 NN in all-pH electrolytes.

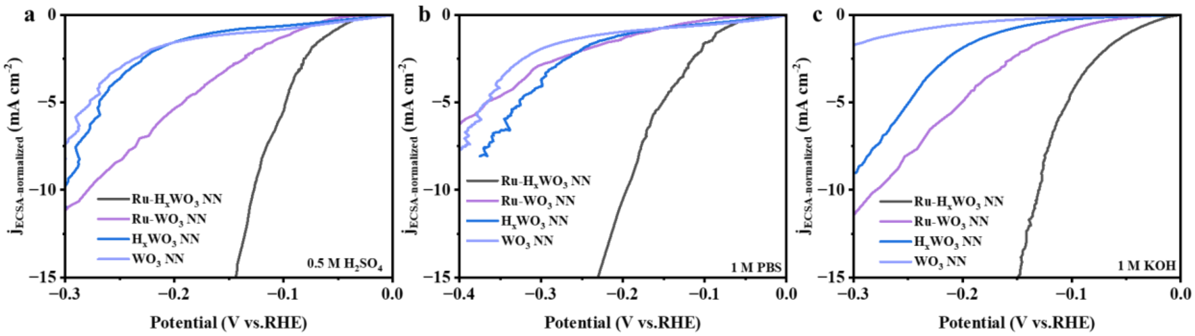

Supplementary Fig. 11. The ECSA-normalized polarization curves. a-c In 0.5 M H_2SO_4 (**a**), 1 M PBS (**b**) and 1 M KOH (**c**). The samples are marked in the figures.

The ECSA-normalized performance of Ru-H_xWO_3 NN is superior to those of WO_3 NN, H_xWO_3 NN and Ru-WO_3 NN in all-pH electrolytes, indicating the highest intrinsic activity of Ru-H_xWO_3 NN among these samples.

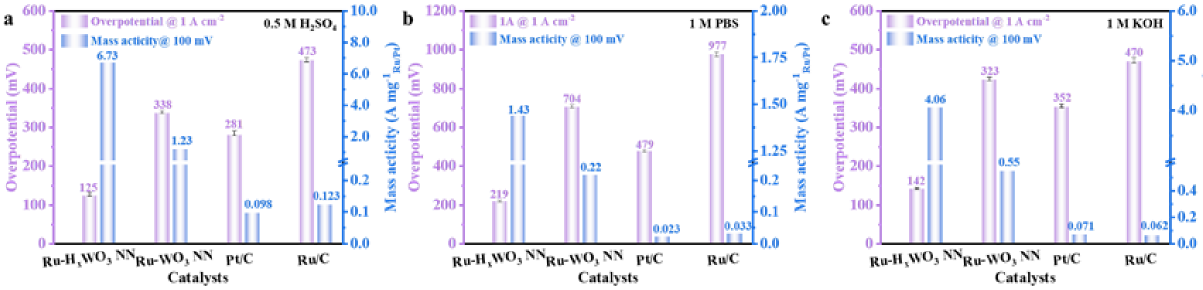

Supplementary Fig. 12. Overpotentials at 1 A cm^{-2} and mass activities at 100 mV. a-c In 0.5 M H_2SO_4 (**a**), 1 M PBS (**b**) and 1 M KOH (**c**). The samples are marked in the figures. **Note:** The error bars for the overpotential at 1 A cm^{-2} are obtained from the polarization curves which is repeatedly tested for at least three times.

Ru-H_xWO_3 NN shows much lower overpotentials at 1 A cm^{-2} and higher mass activities than Ru-WO_3 NN, commercial Pt/C and Ru/C in all-pH electrolytes.

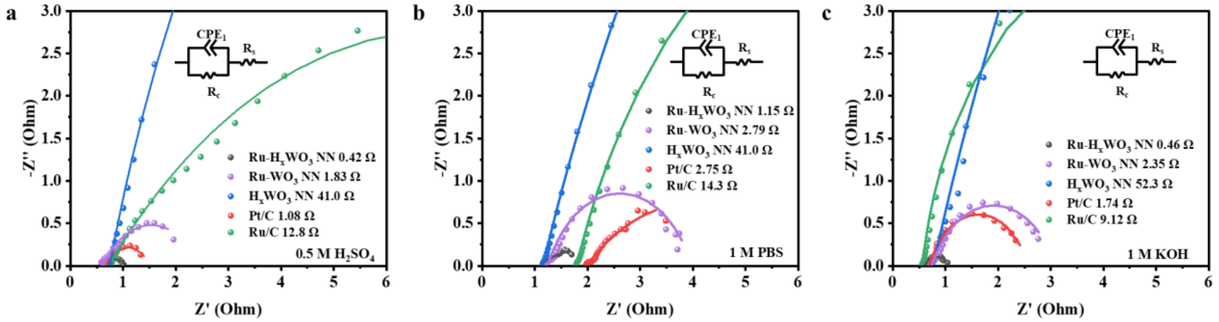

Supplementary Fig. 13. Nyquist plots at HER overpotential of 100 mV. a-c In 0.5 M H_2SO_4 (**a**), 1 M PBS (**b**) and 1 M KOH (**c**). The samples are marked in the figures.

The Ru-H_xWO_3 NN exhibits the smallest charge-transfer resistance (R_{ct}) in all-pH electrolytes among the examined catalysts.

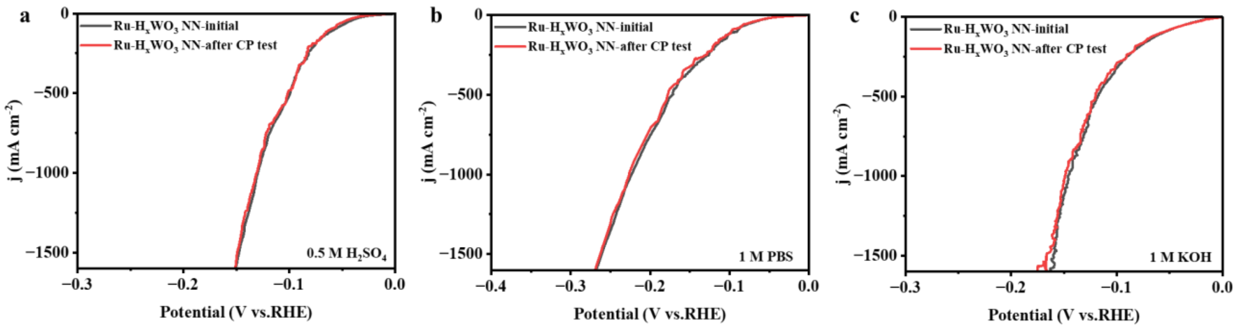

Supplementary Fig. 14. Polarization curves before and after 500 h durability tests. a-c In 0.5 M H_2SO_4 (**a**), 1 M PBS (**b**) and 1 M KOH (**c**).

The LSV curves after durability test exhibit negligible change compared to the initial curves in all-pH electrolytes, indicating extraordinary long-term durability for HER.

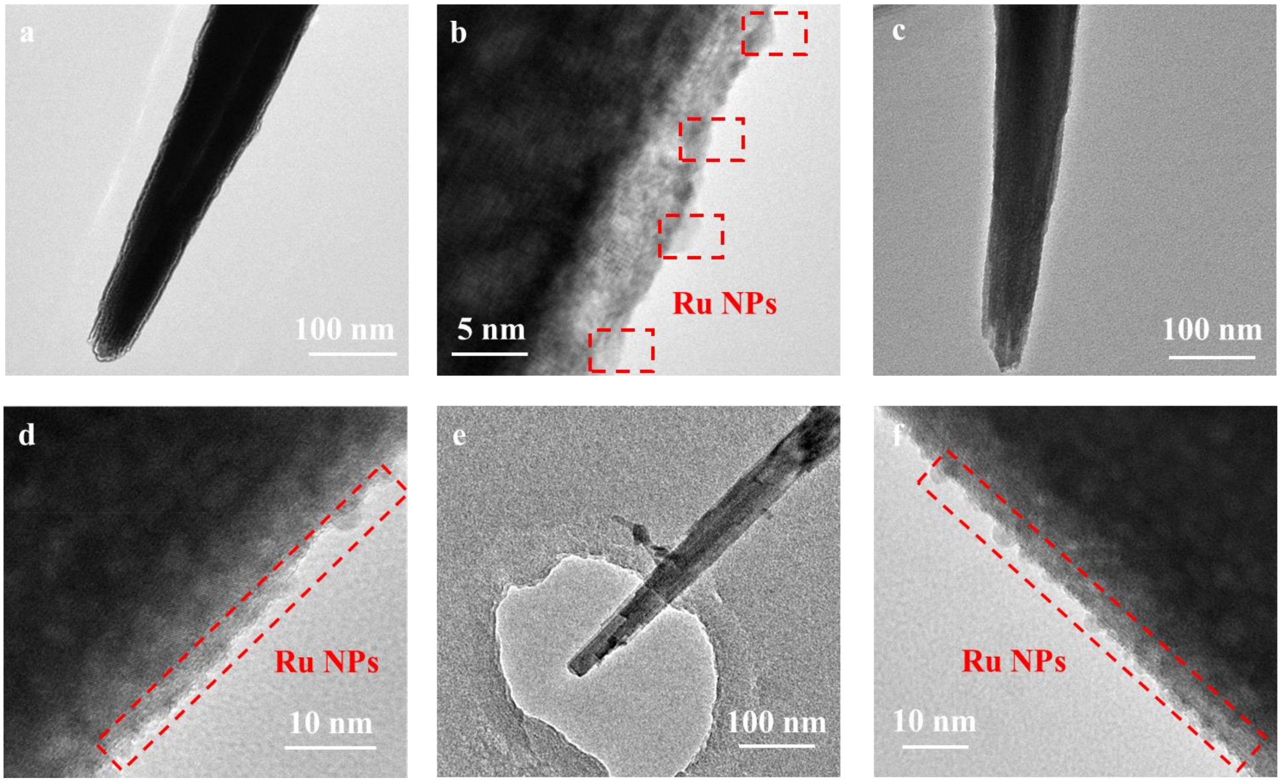

Supplementary Fig. 15. TEM images of Ru-H_xWO_3 NN after the HER tests. a-f In 0.5 M H_2SO_4 (**a,b**), 1 M PBS (**c,d**) and 1 M KOH (**e,f**).

Ru-H_xWO_3 NN shows no significant alterations in its nanoneedle morphology or the size of Ru NPs after the durability test in all-pH electrolytes.

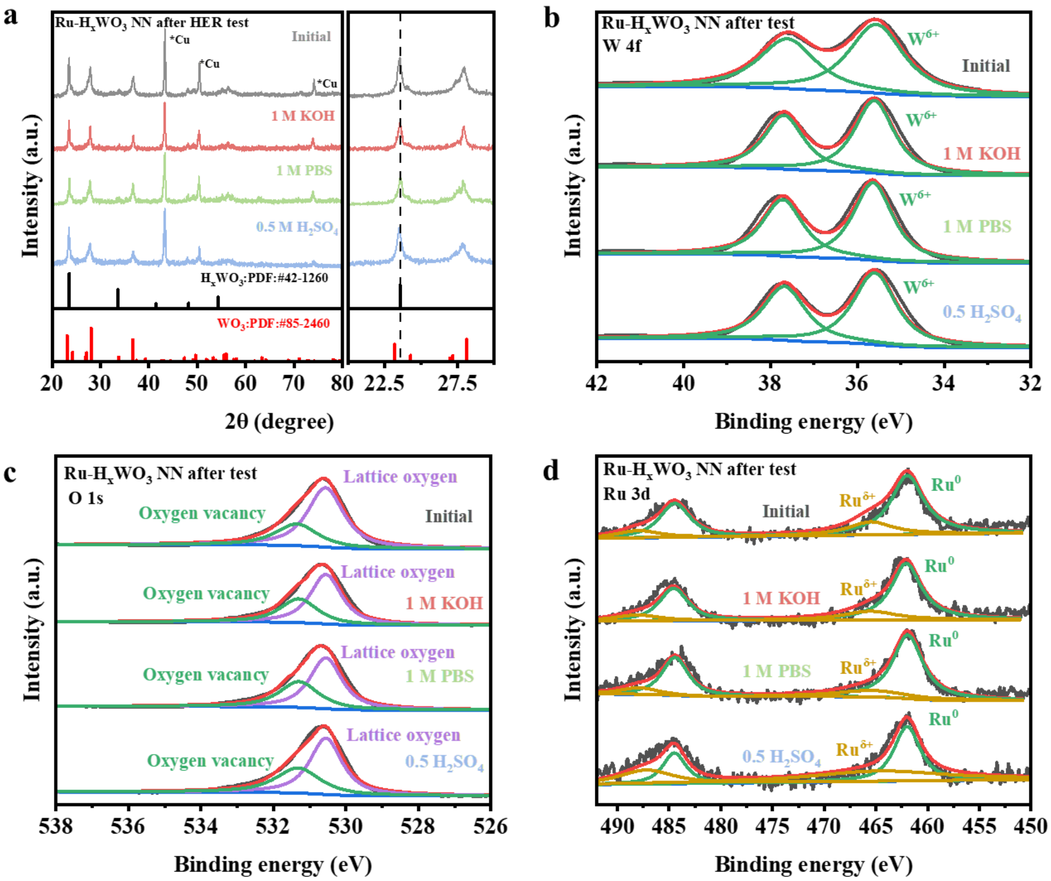

Supplementary Fig. 16. Characterizations of Ru-H_xWO_3 NN before and after the HER durability test. a XRD patterns. **b-d** XPS spectra of W 4*f* (**b**), O 1*s* (**c**), and Ru 3*d* (**d**).

The Ru-H_xWO_3 NN after the durability test shows nearly identical composition and element valences compared to the initial Ru-H_xWO_3 NN, indicating its high stability in all-pH electrolytes.

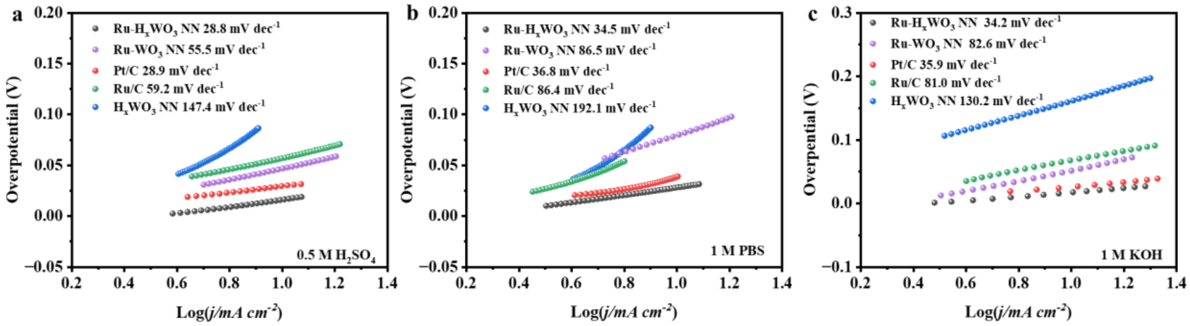

Supplementary Fig. 17. Corresponding Tafel plots. a-c In 0.5 M H_2SO_4 (**a**), 1 M PBS (**b**) and 1 M KOH (**c**). The samples and corresponding Tafel slopes are marked in the figures.

In neutral and alkaline environments, due to the obstruction of proton acquisition and transport, the Tafel slopes tend to be higher than that in acidic environment[11-13].

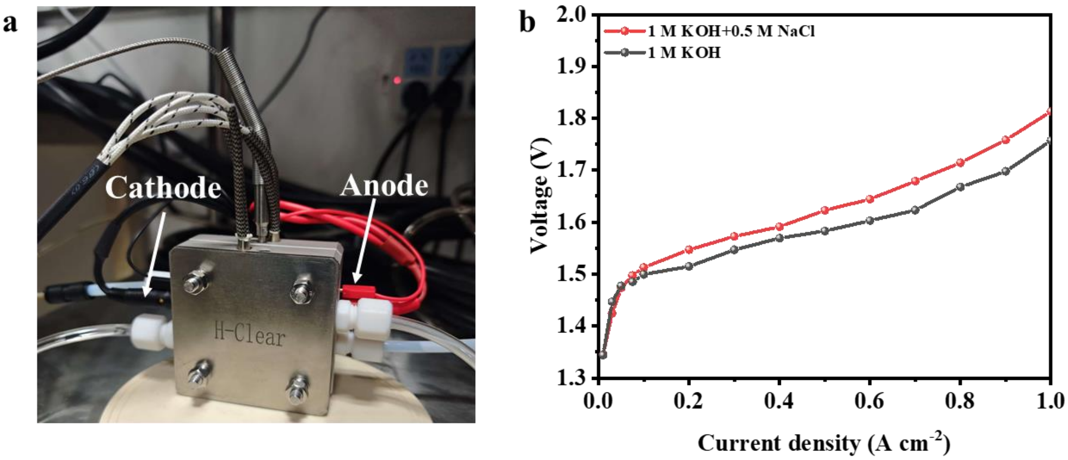

Supplementary Fig. 18. The HER performance of Ru-H_xWO_3 NN in AEMWE test. a The photograph of AEM device. **b** Polarization curves of AEMWE in 1 M KOH or 1 M KOH+0.5 M NaCl.

The HER performance of Ru-H_xWO_3 NN is also evaluated in an anion exchange membrane water electrolyzer (AEMWE) with NiFe-LDH as the anode. To deliver an industrial current density of 1 A cm^{-2}, the cell only requires a low cell voltage of 1.75 V in 1 M KOH and 1.81 V in the simulated alkaline seawater (1 M KOH+0.5 M NaCl).

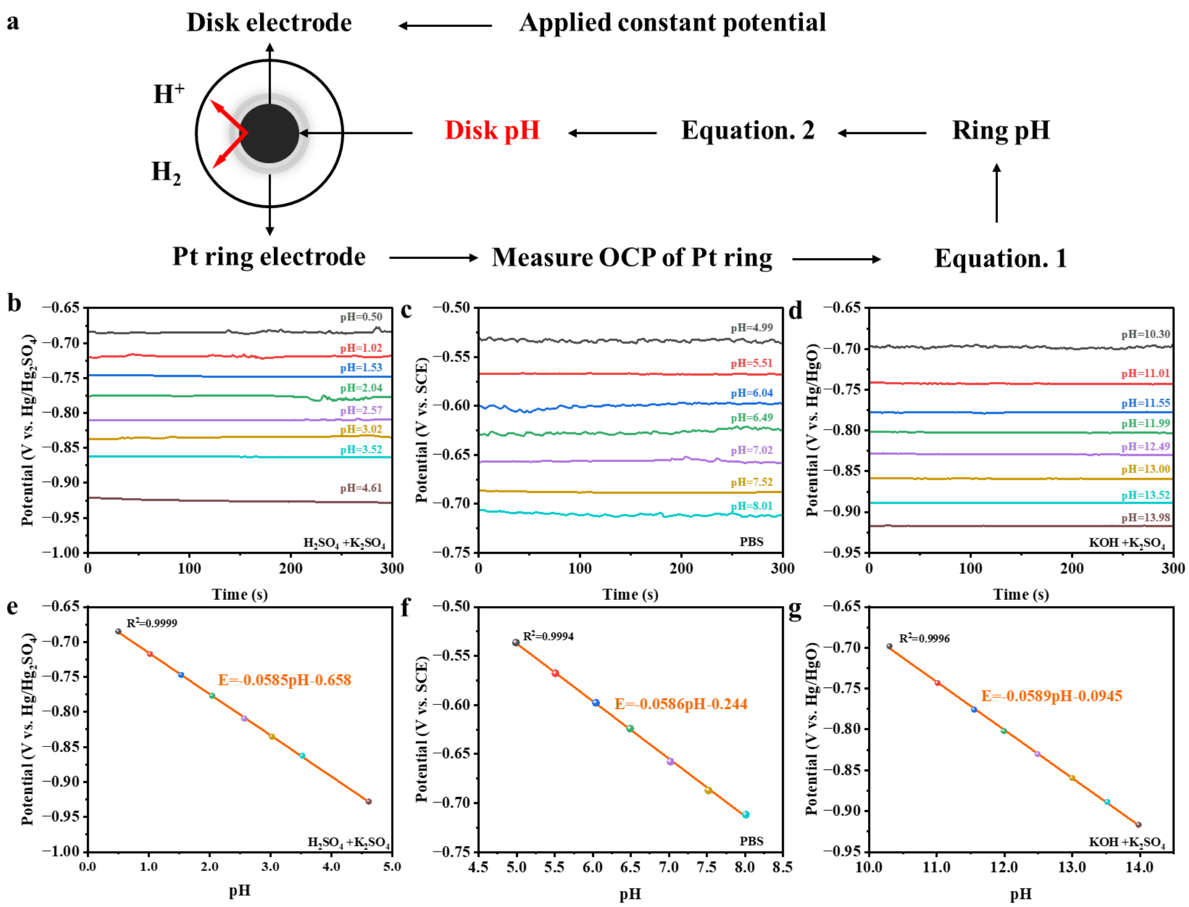

Supplementary Fig. 19. Measurement of local pH value via RRDE technique. a Schematic diagram for monitoring pH on the electrode surface (Equations 1 and 2 are given in Method Section). **b-d** Change of open circuit potential (E_{Rocp}) with time for Pt ring electrode in H_2SO_4 (**b**), PBS (**c**), and KOH (**d**) solution. **e-g** pH dependence of E_{Rocp} for Pt ring electrode in H_2SO_4 (**e**), PBS (**f**), and KOH (**g**) solution.

The RRDE technique was employed to measure the local pH value on the disk electrode (Supplementary Fig. 19a) The corresponding potentials were measured by adjusting the pH value of the electrolytes (Supplementary Fig. 19b-d), and the standard working plots show that there is a linear relationship between the potentials and pH values in all-pH electrolytes (Supplementary Fig. 19e-g).

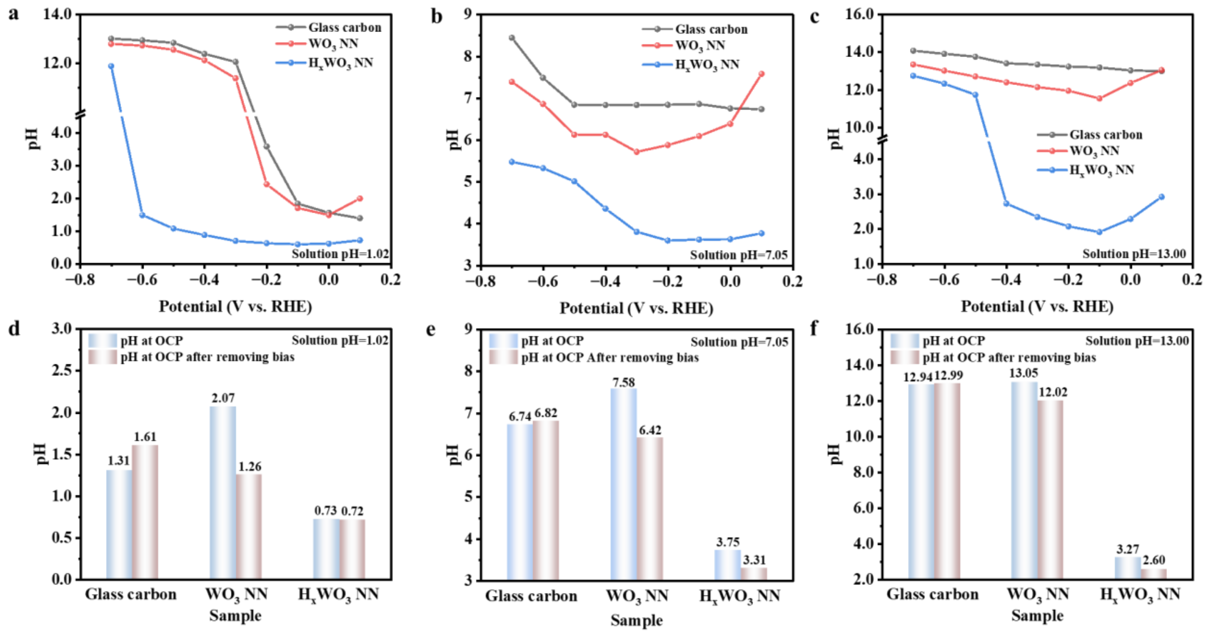

Supplementary Fig. 20. Local pH values on H_xWO_3 NN, WO_3 NN and GC surfaces at different potentials. a-b Measured pH values in H_2SO_4 (**a**), PBS (**b**) and KOH (**c**). **d-f** The pH values before and after removing bias.

The H_xWO_3 NN shows a more acidic environment in 0.1 M H_2SO_4 compared to WO_3 NN and GC. Interestingly, the H_xWO_3 NN can maintain the acidic environment even in 0.1 M KOH. However, the WO_3 NN and GC can only show a pH environment similar to that of the bulk solutions. Notably, the pH values of H_xWO_3 NN and WO_3 NN decrease as the potential is lowered from 0.1 to -0.1 V, which result from the electrochemical proton insertion into the H_xWO_3/WO_3.

Note: It should be pointed out that this pH measurement method is more accurate under neutral conditions [1]. Due to the relatively large value of c_{∞,H^+} or c_{∞,OH^-} in Equation (2) in strongly acidic or alkaline environments, there is the specific OCP of Pt-RE corresponding to pH=7 on DE during testing. Ultrasmall disturbances near the above specific OCP of Pt-RE can cause drastic changes in pH on DE, but this does not affect the trend over a large potential range and the above conclusion was verified in this work.

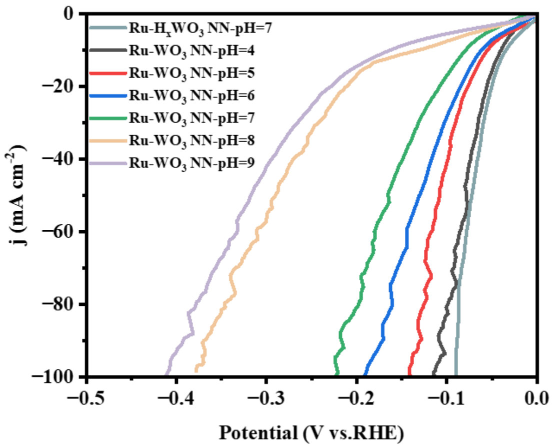

Supplementary Fig. 21. HER polarization curves of Ru-WO_3 NN in weak acid or alkaline electrolytes with different pH.

The HER performance of Ru-WO_3 NN is pH-dependent in near-neutral solutions. Ru-H_xWO_3 NN shows a performance in 1 M PBS (pH=7) similar to that of Ru-WO_3 NN in the PBS electrolyte of pH=4, which implies that the H_xWO_3 NN can create an acidic environment with the pH < 4 even in 1 M PBS.

a **b**

c **d**

e

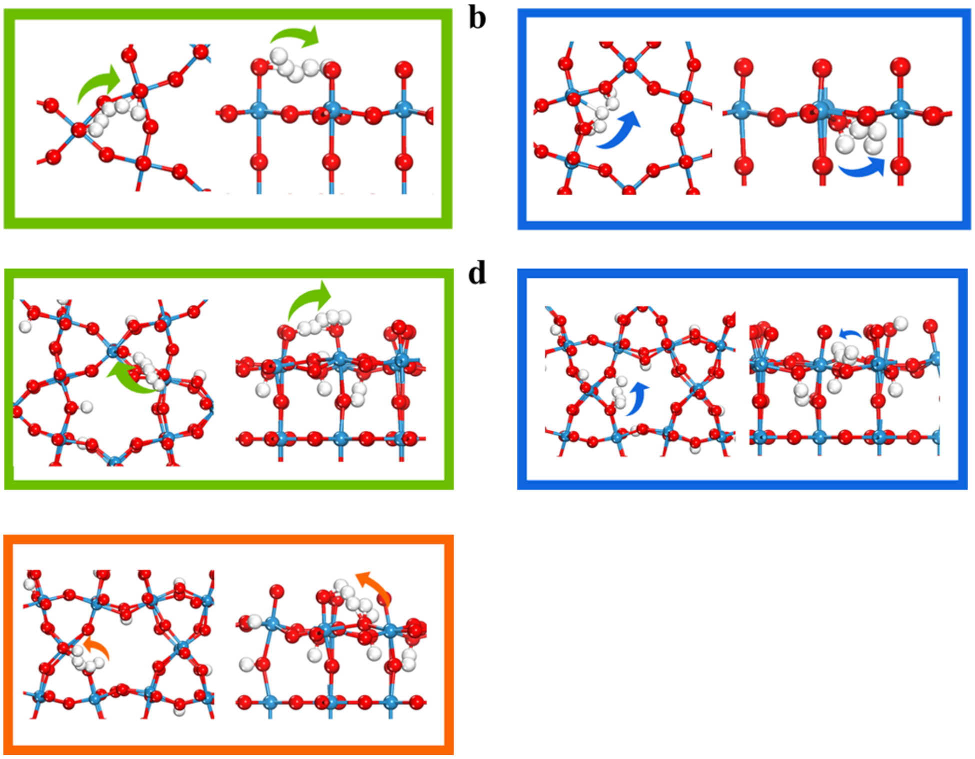

Supplementary Fig. 22. The models for lattice-H migration. a,b Lattice-H migration between adjacent $O_{Terminal}$ sites (**a**) and $O_{Bridging}$ sites (**b**) in WO_3 support. **c,d** Lattice-H migration between adjacent $O_{Terminal}$ sites (**c**) and $O_{Bridging}$ sites (**d**) in H_xWO_3 support. **e** Lattice-H migration from $O_{Bridging}$ to $O_{Terminal}$ sites in H_xWO_3 support. The left and right of each image is the top view and side view, respectively.

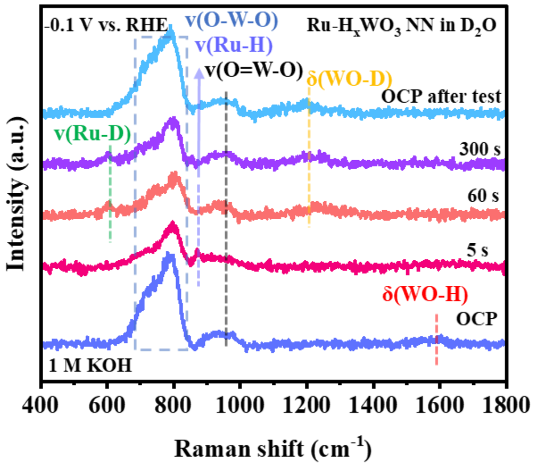

Supplementary Fig. 23. ***In situ*** **Raman spectra of Ru-H_xWO_3 NN at -0.1 V vs. RHE in 1 M KOH D_2O solution at different stages of HER.**

In 1 M KOH D_2O solution, the Ru-H peak (~872 cm^{-1}) appears at the beginning of HER (e.g., 5 s) and then is replaced by the Ru-D peak (~600 cm^{-1}) as the HER progresses (e.g., 60 s and 300 s). In addition, the WO-H peak (~1580 cm^{-1}) is gradually replaced by the WO-D peak (~1200 cm^{-1}) during HER. This result confirms the dynamic migration and replenishment of lattice-H/D.

Note: The *in situ* Raman spectra test of the isotope exchange experiment wasn't conducted in acidic media due to the disturbance of H_2SO_4.

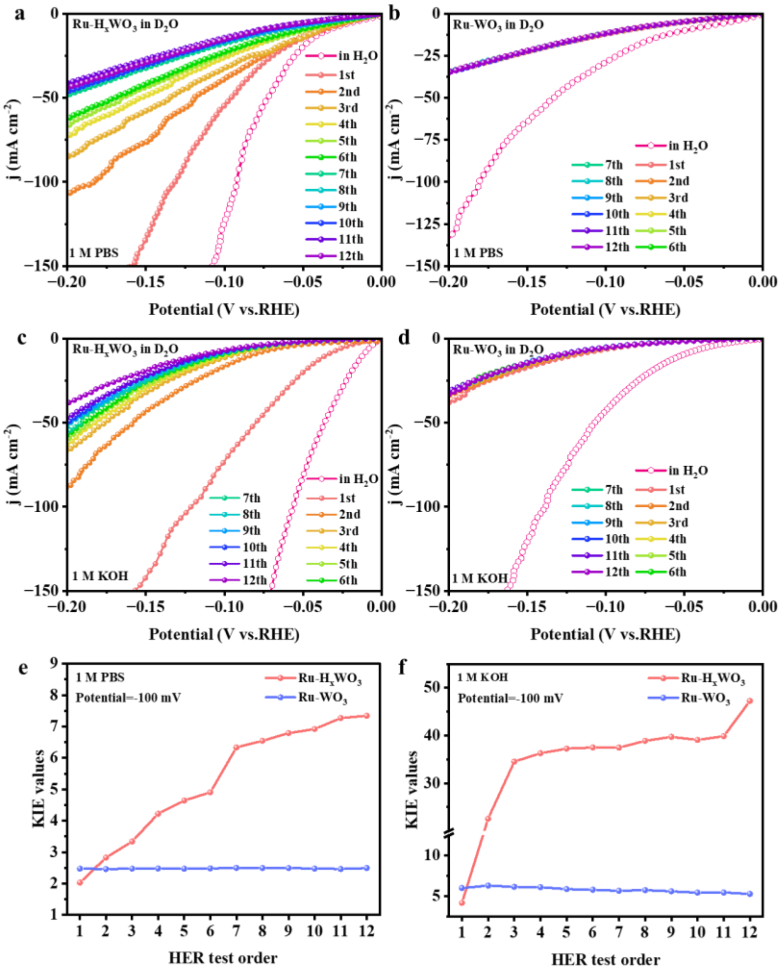

Supplementary Fig. 24. KIE experiments. a,b LSV curves of Ru-H_xWO_3 (**a**) and Ru-WO_3 (**b**) in 1 M PBS (H_2O and D_2O) solutions; **c,d** LSV curves of Ru-H_xWO_3 (**c**) and Ru-WO_3 (**d**) in 1 M KOH (H_2O and D_2O) solutions; **e,f** Calculated KIE values (J_{H2O}/J_{D2O}) at 100 mV in 1 M PBS (**e**) and 1 M KOH (**f**).

The performances of catalysts in alkaline/neutral D_2O solution were tested by the same LSV measurement described previously. In PBS solution, both Ru-H_xWO_3 and Ru-WO_3 NN showed poorer HER performance in the D_2O electrolyte than that in the H_2O electrolyte due to the inactivity of the D element in D_2O solution (Supplementary Fig. 24a,b). In the D_2O electrolyte, the LSV curves of Ru-WO_3 NN remained unchanged across different scan numbers. In contrast, the LSV curves of Ru-H_xWO_3 NN showed a progressive negative shift in potential as the scan number increased. Similar phenomena were also observed in 1 M KOH electrolytes using either H_2O or D_2O as the solvents (Supplementary Fig. 24c,d).

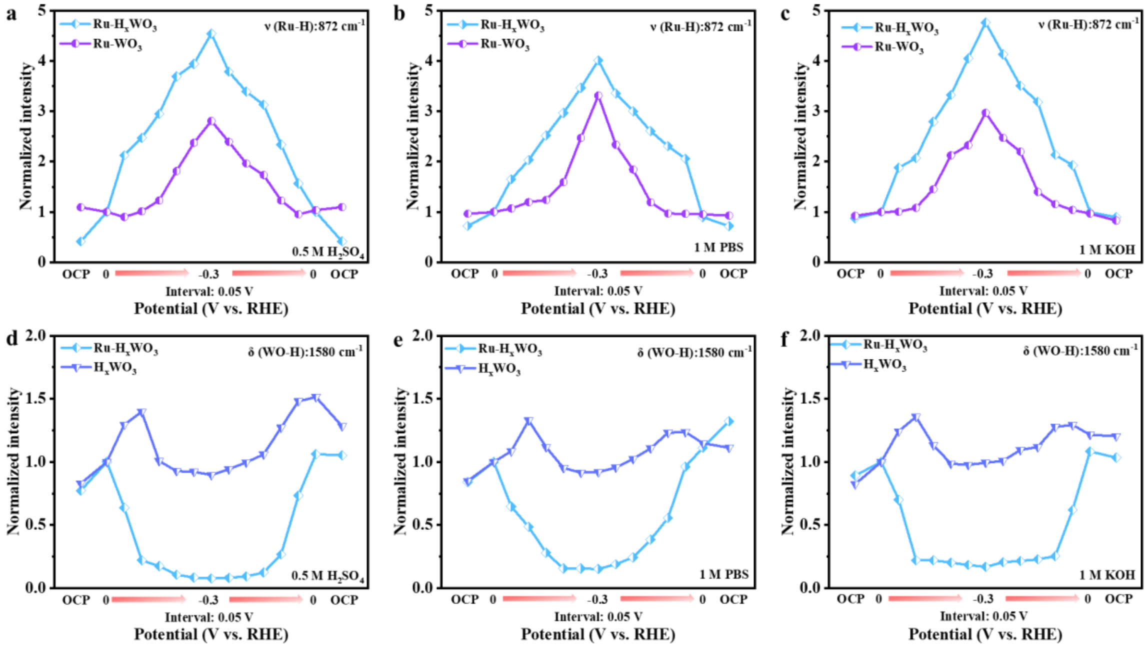

Supplementary Fig. 25. The intensity difference of Ru-H and WO-H peaks at various potentials. **a-c** Normalized intensity of Ru-H peak from OCP to -0.3 V in 0.5 M H_2SO_4 (**a**), 1 M PBS (**b**) and 1 M KOH (**c**). **d-f** Normalized intensity of WO-H peak from OCP to -0.3 V in 0.5 M H_2SO_4 (**d**), 1 M PBS (**e**) and 1 M KOH (**f**).

The intensities of Ru-H peak for Ru-H_xWO_3 NN are significantly stronger than that for Ru-WO_3 NN in all-pH electrolytes, indicating the easier lattice-H migration from H_xWO_3 NN to Ru sites. In H_xWO_3, the strengthening of WO-H peak in the range of OCP to -0.1 V is due to electrochemical insertion of H. The subsequent decrease of WO-H peak in the range of -0.1 V to -0.3 V is due to the consumption of H species during HER. The WO-H peak weakens while the Ru-H peak increases as the potential becomes more negative in all-pH electrolytes for the Ru-H_xWO_3 NN.

The method of normalization: the peak intensity of 0 V vs. RHE was set as the reference peak intensity, and then calculate the ratio of the peak intensity at other potentials to the reference peak intensity.

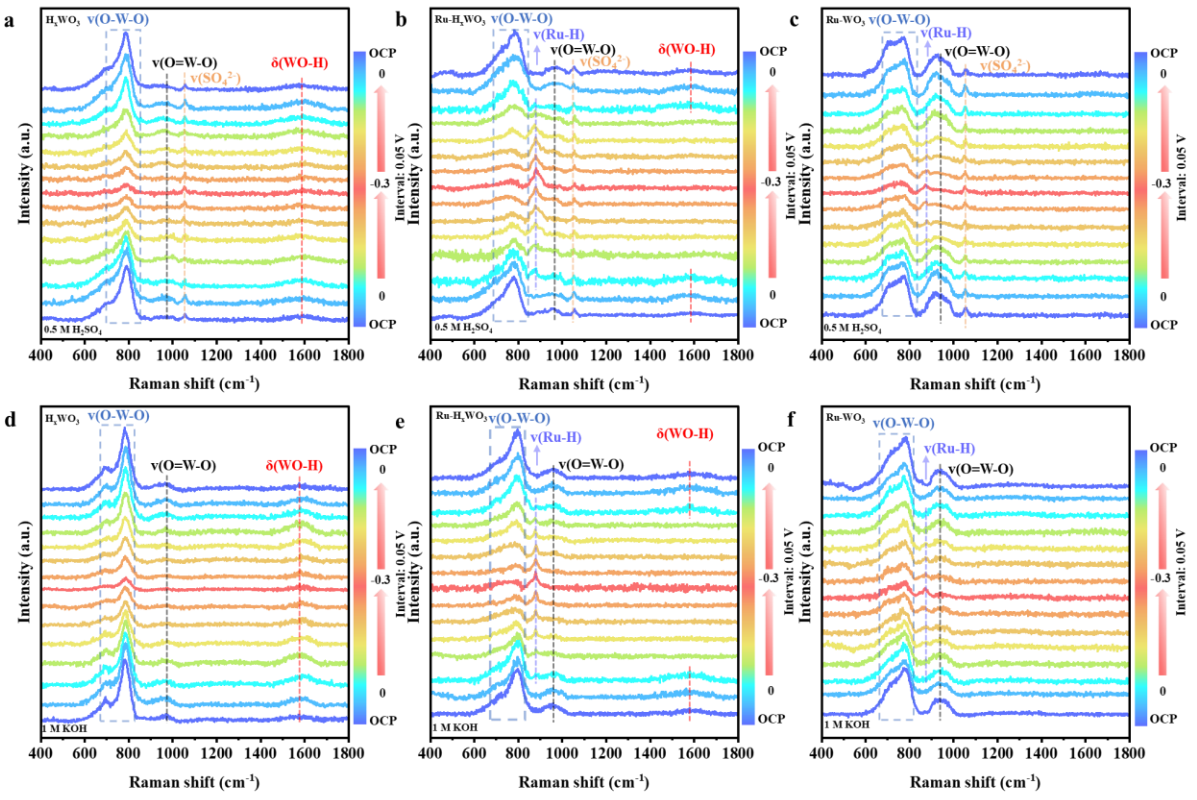

Supplementary Fig. 26. *In situ* Raman spectra from OCP to -0.3 V. a-c H_xWO_3 (**a**), Ru-H_xWO_3 (**b**), and Ru-WO_3 (**c**) NN in 0.5 M H_2SO_4. **d-f** H_xWO_3 (**d**), Ru-H_xWO_3 (**e**), and Ru-WO_3 (**f**) NN in 1 M KOH. Note: In 0.5 M H_2SO_4 solution, the Raman peak at ~1050 cm^{-1} is associated to the symmetric stretching mode of SO_4^{2-}.

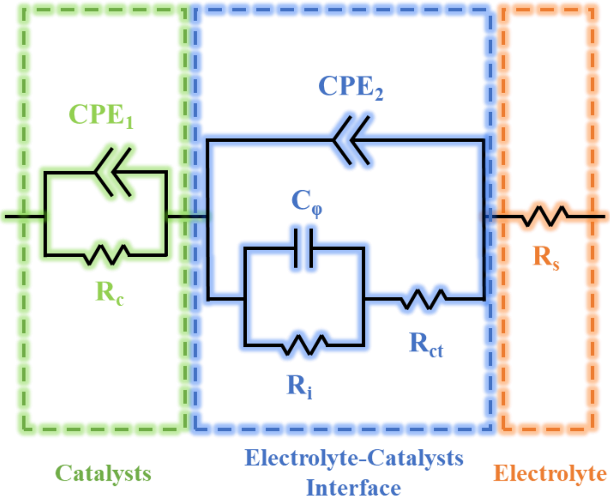

Supplementary Fig. 27. Schematic diagram of the equivalent circuit.

The first part (green part) contains a resistor (R_c) and a constant phase element (CPE_1). The R_c and CPE_1 correspond to the resistance and the double-layer capacitance in inner layer of catalyst, respectively. The second part (blue part) corresponds to the charge transfer processes (R_{ct} and CPE_2) and intermediate accumulation processes (R_i and $C_φ$) at the electrolyte-catalyst interface. CPE_2 and $C_φ$ represents the double-layer capacitance and the hydrogen adsorption pseudo-capacitance at the electrolyte-catalyst interface, respectively. R_c, R_{ct}, and R_i refer to charge-transfer resistance in the corresponding double-layer capacitors and pseudo-capacitors, respectively. The third part (orange part) corresponds to the internal resistance of electrolyte and totally circuit (R_s), which is usually constant with the increase of potential.

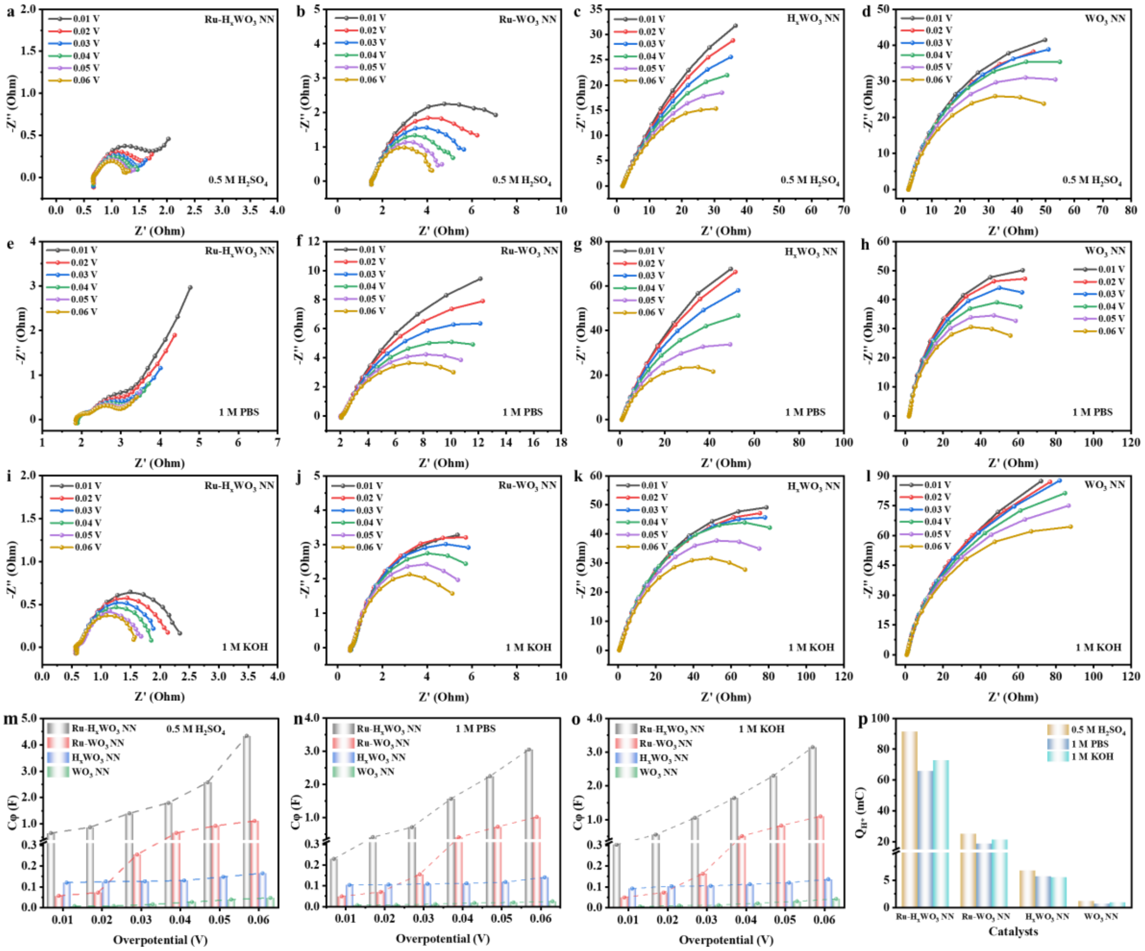

Supplementary Fig. 28. Measurement of C_φ and Q_{H^*} of Ru-H_xWO_3, Ru-WO_3, H_xWO_3, and WO_3 NN. **a-l** Nyquist plots at different overpotentials in different electrolytes. **m-o** Fitted data of C_φ at different overpotentials in 0.5 M H_2SO_4 (**m**), 1 M PBS (**n**) and 1 M KOH (**o**). **p** Integrated Q_{H^*} in 0.5 M H_2SO_4, 1 M PBS and 1 M KOH. The samples and electrolytes are marked in the figures.

The H adsorption behaviors at different overpotentials were further explored through in situ EIS measurements (Supplementary Fig. 28a-l). Based on electrolyte-catalyst interface, the adsorption pseudo capacitance of H^* (C_φ) can be integrated as a function of overpotential, which can be used to calculate the hydrogen adsorption charge (Q_{H^*}) to learn the H* coverage on the catalyst [14] (Supplementary Fig. 27). The value of C_φ at nearly zero potential can reflect the intrinsic hydrogen coverage before HER. In our study, the H-containing samples of H_xWO_3 NN and Ru-H_xWO_3 NN showed higher initial C_φ values (at 0.01 V) than the H-free samples of WO_3 NN and Ru-WO_3 NN in all-pH electrolytes due to the presence of lattice-H in the former (Supplementary Fig. 28m-o). Based on the C_φ values at different potentials (0.01-0.06 V), the calculated Q_{H^*} of Ru-H_xWO_3 NN is much larger than those of the other three samples (Supplementary Fig. 28p). This result indicates that the coupling of Ru and H_xWO_3 NN significantly enhances the H^* coverage. The substantially higher H^* coverage in Ru-H_xWO_3 NN than that in Ru-WO_3 NN accounts for the ultralow HER overpotential of the former.

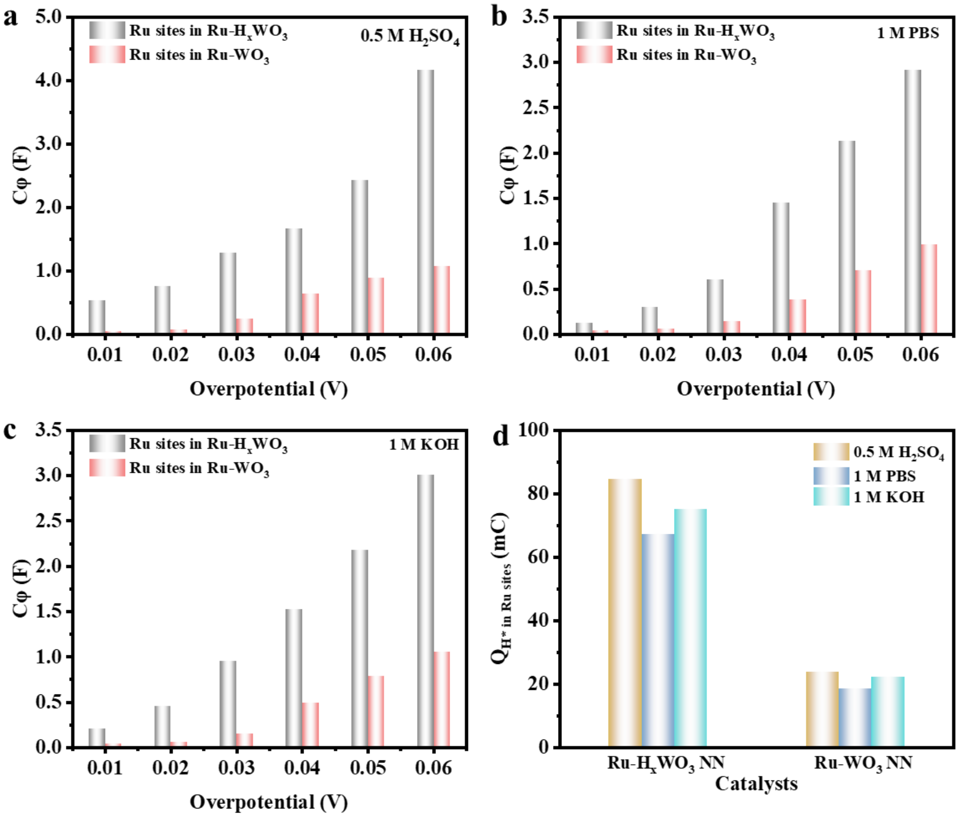

Supplementary Fig. 29. C$_\varphi$ and Q$_{H*}$ for Ru sites of Ru-H$_x$WO$_3$, Ru-WO$_3$ NN. a-c Fitted data of C$_\varphi$ in 0.5 M H_2SO_4 (**a**), 1 M PBS (**b**) and 1 M KOH (**c**). **d** Integrated Q$_{H*}$ in 0.5 M H_2SO_4, 1 M PBS and 1 M KOH.

By further calculating Q$_{H*}$ at Ru sites, i.e., $Q_{H*(Ru)} = Q_{H*(Ru\text{-}support)} - Q_{H*(support)}$, to eliminate the contribution of the support itself, it is found that the Ru sites on Ru-H$_x$WO$_3$ NN still exhibited a much higher H* coverage than those on Ru-WO$_3$ NN. This result indicates that the thermally-hydrogenated H$_x$WO$_3$ support can more effectively migrate lattice-H to create a H*-rich microenvironment at Ru sites, thereby enhancing the HER performance of Ru-H$_x$WO$_3$ NN.

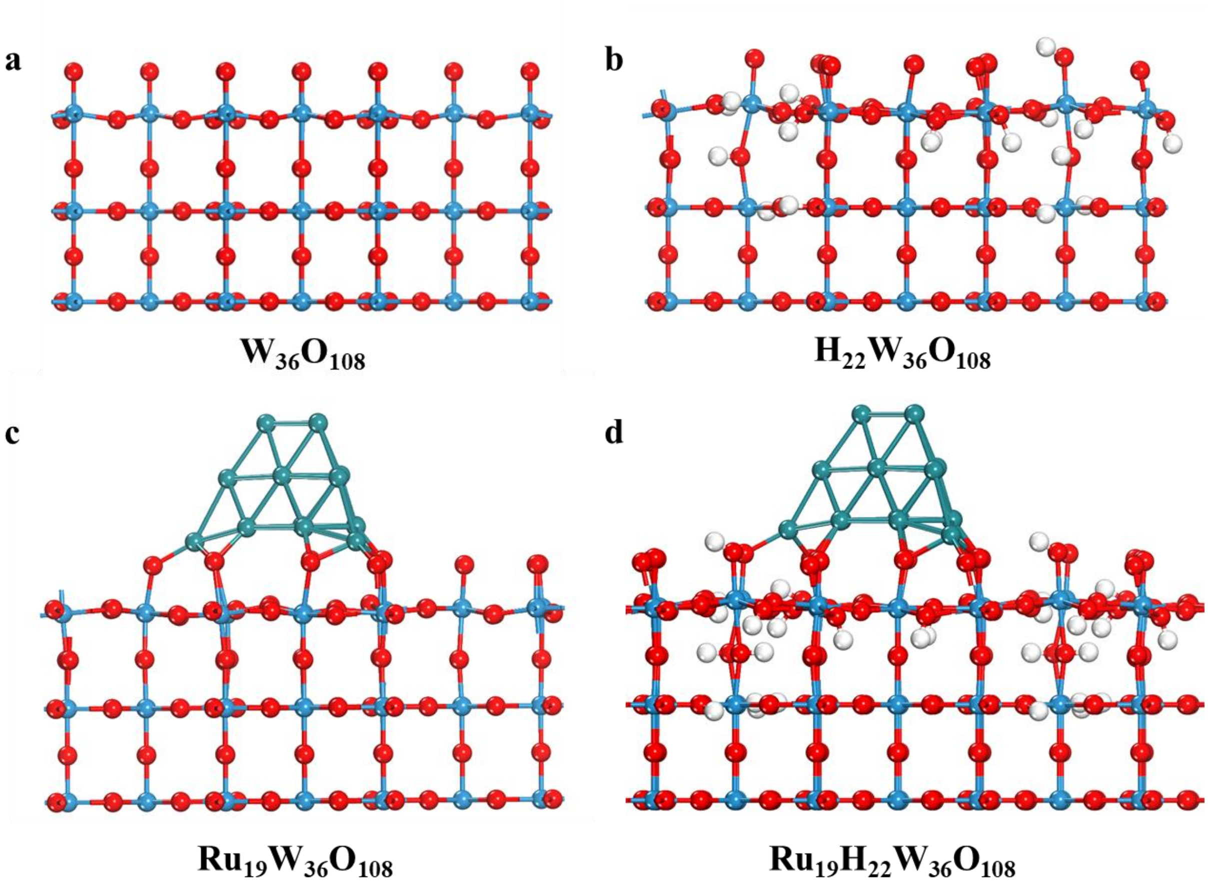

Supplementary Fig. 30. The side illustrations of models for DFT calculation. a WO_3. **b** H_xWO_3. **c** Ru-WO_3. **d** Ru-H_xWO_3.

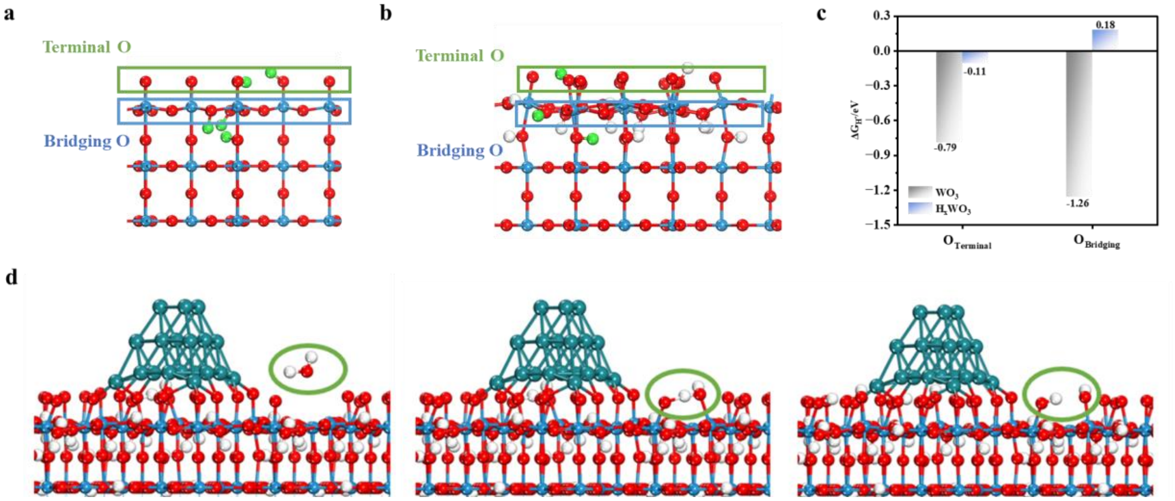

Supplementary Fig. 31. Calculation of H adsorption energy. a,b Schematic diagram of different sites for H adsorption in WO_3 (**a**) and H_xWO_3 (**b**). **c** Corresponding adsorption free energy in WO_3 and H_xWO_3. **d** The process of H_2O molecule dissociation on W sites in Ru-H_xWO_3.

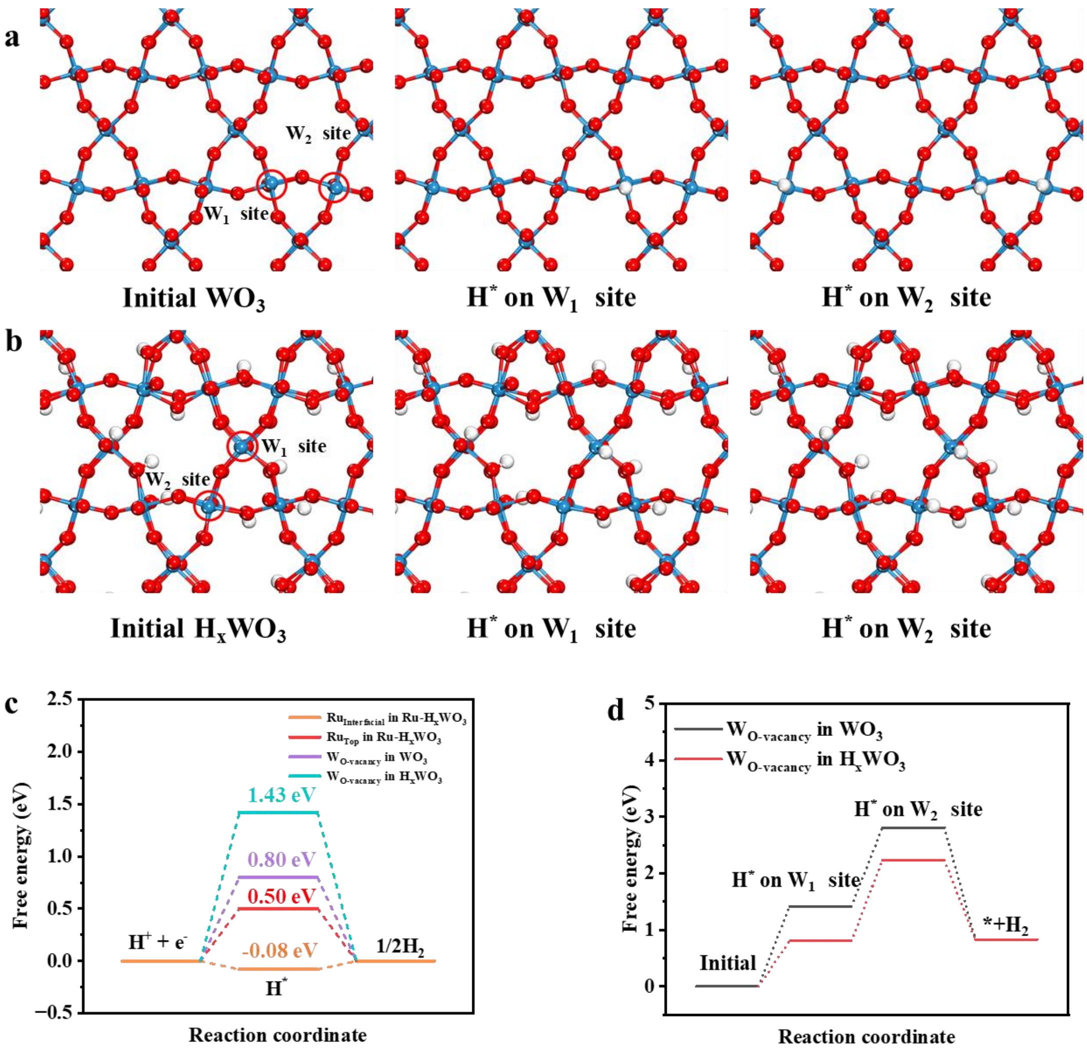

Supplementary Fig. 32. Calculation for H adsorbed on W sites. **a** WO_3. **b** H_xWO_3. **c** Corresponding free energy profiles for HER on single W sites. **d** Corresponding free energy profiles for HER on adjacent W sites.

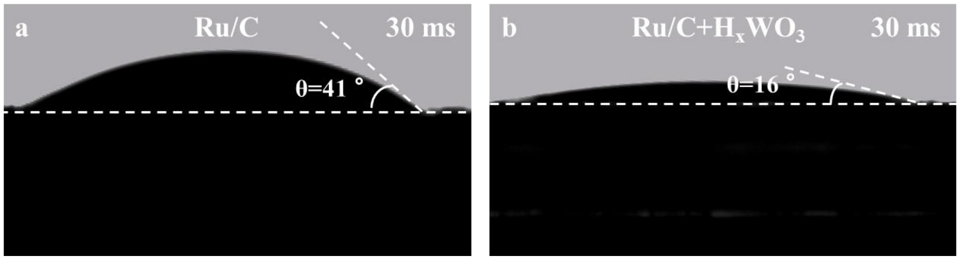

Supplementary Fig. 33 Contact angles of water on a Ru/C and **b** Ru/C+H_xWO_3.

The water contact angles of Ru/C catalyst and Ru/C+H_xWO_3 mixed catalyst are 41° and 16°, respectively, which shows the better adsorption capability of water molecules on the W sites than the case on the Ru sites. **Note**: Ru/C+H_xWO_3 mixed catalyst was prepared by mixing finely ground H_xWO_3 powder with commercial Ru/C catalyst with a mass ratio of 1:1.

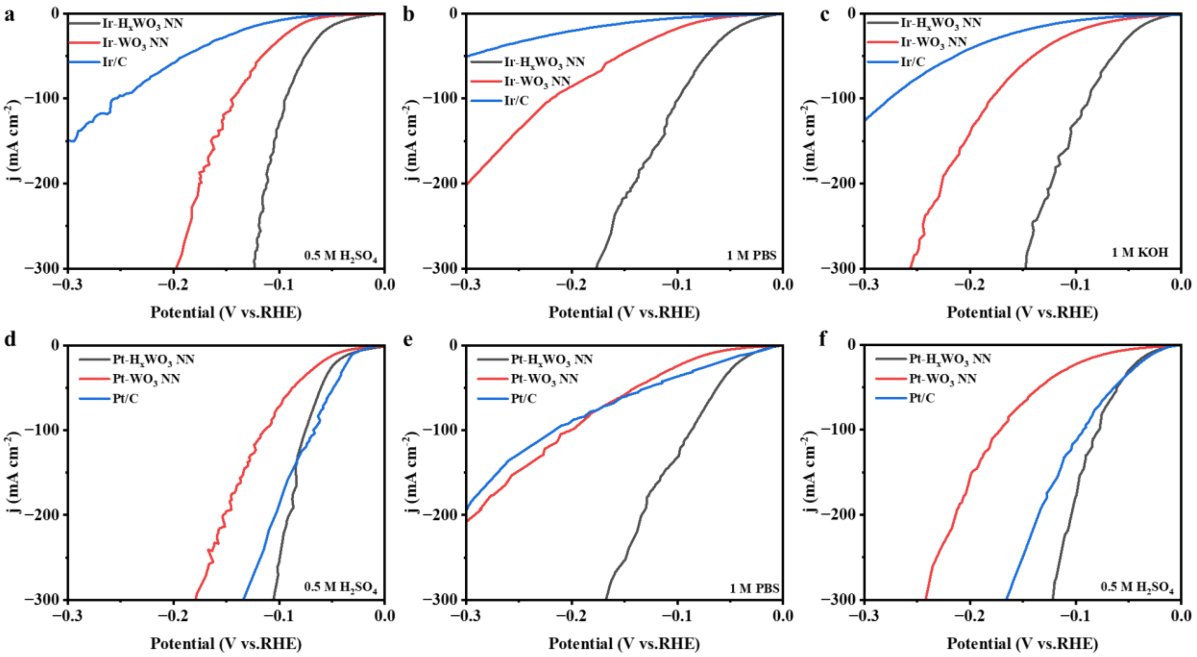

Supplementary Fig. 34. Polarization curves. a-c LSV curves of Ir-H$_x$WO$_3$ NN and Ir-WO$_3$ NN in 0.5 M H_2SO_4 (**a**), 1 M PBS (**b**) and 1 M KOH (**c**). **d-f** LSV curves of Pt-H$_x$WO$_3$ NN and Pt-WO$_3$ NN in 0.5 M H_2SO_4 (**d**), 1 M PBS (**e**) and 1 M KOH (**f**).

Ir-H$_x$WO$_3$ NN and Pt-H$_x$WO$_3$ NN shows the much better performances than Ir-WO$_3$ NN and Pt-WO$_3$ NN, respectively, in all-pH electrolytes.

Supplementary Table S1. HER performances of state-of-the-art pH-universal HER catalysts.

Catalysts	Electrolyte	η_{HER} @10 mA cm^{-2}	η_{HER} @500 mA cm^{-2}	η_{HER} @1A cm^{-2}	Durability	Loading mass of noble metal	Support	Reference
**Ru-H_xWO_3 NN**	**0.5 M H_2SO_4<br>1 M PBS<br>1 M KOH**	**12 mV<br>28 mV<br>14 mV**	**97 mV<br>170 mV<br>114 mV**	**125 mV<br>219 mV<br>142 mV**	**>500 h@1 A cm^{-2}<br>(All pH)**	**1.26 wt.%**	**Cu foam**	**This work**
**RuMo@MoO_x-JH**	0.5 M H_2SO_4<br>1 M PBS<br>1 M KOH	15 mV<br>18 mV<br>9 mV	~110 mV<br>~325 mV<br>~ 110 mV	~160 mV<br>/<br>169 mV	2000 h@1 A cm^{-2}<br>(1 M KOH)	136<br>μg cm^{-2}	Ni foam	12
**Ru–PtFeNiCuW /CNTs**	0.5 M H_2SO_4<br>1 M PBS<br>1 M KOH	9 mV<br>53 mV<br>16 mV	/	/	50 h@10 mA cm^{-2}<br>(alkaline and acid)	/	Glassy carbon	15
**RuCo@NC-600**	0.5 M H_2SO_4<br>1 M PBS<br>1 M KOH	6 mV<br>60 mV<br>34 mV	/	/	120 h@10 mA cm^{-2}<br>(1 M KOH)	/	Glassy carbon	16
**Ir/HfO_2@C**	0.5 M H_2SO_4<br>1 M PBS<br>1 M KOH	18 mV<br>51 mV<br>28 mV	~200 mV<br>/<br>~260 mV	/	550 h@10 mA cm^{-2}<br>(alkaline and acid)	/	Carbon cloth	17
**$Rh(OH)_3$/$Co(OH)_2$**	0.5 M H_2SO_4<br>1 M PBS<br>1 M KOH	12 mV<br>25 mV<br>13 mV	/	/	70 h@10 mA cm^{-2}<br>(alkaline and acid)	0.02<br>mg cm^{-2}	Carbon paper	18
**$NiRu_{0.13}$-BDC**	1 M HCl<br>1 M PBS<br>1 M KOH	13 mV<br>36 mV<br>34 mV	/	/	30 h@50 mA cm^{-2}<br>(1 M PBS)	/	Ni foam	11
**Ru_n-NPO**	0.5 M H_2SO_4<br>1 M PBS<br>1 M KOH	23 mV<br>35 mV<br>36 mV	/	/	200 h@10 mA cm^{-2}<br>(All pH)	2.63 wt%	Carbon cloth	19
**$RuSe_{1.5}$ NPs**	0.5 M H_2SO_4<br>1 M PBS<br>1 M KOH	24 mV<br>30 mV<br>12 mV	/	/	22 h@10 mA cm^{-2}<br>(1 M KOH)	/	Glassy carbon	20
**Ru/V-NHWO**	0.5 M H_2SO_4<br>1 M PBS<br>1 M KOH	30 mV<br>41 mV<br>28 mV	/	/	20 h@10 mA cm^{-2}<br>(All pH)	0.44 wt%	Carbon cloth	21
**Ru_{SA}@NiFe PPc**	0.5 M H_2SO_4<br>1 M PBS<br>1 M KOH	40 mV<br>350 mV<br>12 mV	/	/	45 h@100 mA cm^{-2}<br>(alkaline and acid)	0.7 wt%	Glassy carbon	22
**Ru/MoO_2@NCF**	0.5 M H_2SO_4<br>1 M PBS<br>1 M KOH	42 mV<br>70 mV<br>9 mV	/	/	50 h@10 mA cm^{-2}<br>(All pH)	≈7 wt%	Glassy carbon	23
**Ru_1CoP/CDs**	0.5 M H_2SO_4<br>1 M KOH	49 mV<br>51 mV			20 h@10 mA cm^{-2}<br>(alkaline and acid)	/	Glassy carbon	24
**Ru-CoP/Ni_2P**	0.5 M H_2SO_4<br>1 M PBS<br>1 M KOH	53 mV<br>125 mV<br>64 mV	/	/	50 h@100 mA cm^{-2}<br>(All pH)	33.1<br>μg cm^{-2}	Ni foam	25

Note: Most of the reported pH-universal catalysts for HER typically show single high performance in either acidic or alkaline electrolyte, and suffer a much high overpotential in neutral electrolyte due to the different HER mechanism in different pH. In addition, these catalysts are difficult to work at industrial-level current densities (@>500 mA cm^{-2}), and the durability of these catalysts

is usually <100 h at low current densities (<100 mA cm^{-2}), failing to meet the demand of practical application. Thanks to the lattice-H cycling mechanism, the Ru-H_xWO_3 NN show excellent HER performances and ultrahigh durability (>500 h@1 A cm^{-2}) in all-pH electrolytes even at industrial-level current densities.

Supplementary Table S2. HER performances of state-of-the-art HER catalysts at 10 mA cm^{-2} and 1 A cm^{-2}.

Catalysts	Electrolyte	η_{HER} @10 mA cm^{-2}	η_{HER} @1A cm^{-2}	Durability	Loading mass of noble metal	Support	Reference
**Ru-H_xWO_3 NN**	**0.5 M H_2SO_4<br>1 M PBS<br>1 M KOH**	**12 mV<br>28 mV<br>14 mV**	**125 mV<br>219 mV<br>142 mV**	**>500 h@1 A cm^{-2} (All pH)**	**1.26 wt.%**	**Cu foam**	**This work**
MoO_2@Ru NT	1 M KOH	22 mV	131 mV	100 h@1 A cm^{-2} (1 M KOH)	158 μg cm^{-2}	Carbon paper	26
Ru/(Fe,Ni)$(OH)_2$	1 M KOH	13 mV	152 mV	20 h@1 A cm^{-2}	/	Ni foam	27
RH-Ni_9S_8/RuS_2	1 M KOH	38 mV	180 mV	500 h@1 A cm^{-2} (1 M KOH)	/	Ni foam	28
$CdNNi_3$	1 M KOH	65 mV	235 mV	400 h@1 A cm^{-2} (1 M KOH)	/	Ni foam	29
UP-$RuNi_{SAs}$/C	1 M KOH	9 mV	253 mV	100 h@>1 A cm^{-2} (1 M KOH)	/	Carbon paper	30
Fe_2P−Co_2P	1 M KOH	81 mV	254 mV	90 h@1 A cm^{-2}	/	Cu foam	31
WS_2 superstructure	1 M KOH	/	264 mV	100 h@>1 A cm^{-2} (1 M KOH)	/	Glassy carbon	32
IrNi-$FeNi_3$	1 M KOH	31 mV	289 mV	124 h@1 A cm^{-2}	1.218 wt.%	Ni foam	33
Ni–P–B	1 M KOH	/	345 mV	50 h@ 1 A cm^{-2}	/	Filter paper	34
Am-NiMoB	1 M PBS<br>1 M KOH	38 mV<br>48 mV	778 mV<br>283 mV	30 h@500 mA cm^{-2} (1 M PBS)	/	Ni foam	35
Pd/Ir Hetero-Metallene	0.5 M H_2SO_4	21 mV	133 mV	100 h@250 mA cm^{-2}	/	Glassy carbon	36
p-Pt_3V	0.5 M H_2SO_4	20 mV	300 mV	100 h@500 mA cm^{-2}	/	Carbon cloth	37
Co-N-C	0.5 M H_2SO_4	/	343 mV	32 h@ 1 A cm^{-2}	/	Carbon film	38
Co/Se-MoS_2	0.5 M H_2SO_4	104 mV	382 mV	360 h@ 1 A cm^{-2}	/	Ni foam	39
Sr_2RuO_4	0.5 M H_2SO_4<br>1 M KOH	18 mV<br>28 mV	182 mV<br>278 mV	800 h@1 A cm^{-2} (1 M KOH)	/	Cu wire	40

Note: Ru-H_xWO_3 NN show an excellent HER performance and ultrahigh durability (>500 h@1 A cm^{-2}), which are better than most of HER catalysts reported in single electrolyte.

Supplementary Table S3. Element content of Ru-H_xWO_3 NN and Ru-WO_3 NN by XPS analysis.

Sample \ Content	W /at.%	O /at.%	Ru /at.%	Ru /wt.%
**Ru-H_xWO_3 NN**	13.7	58.61	0.42	1.21
**Ru-WO_3 NN**	13.1	53.11	0.47	1.26

Note: C 1*s* is used to calibrate XPS peak and the content of C is not listed in this table.

Supplementary Table S4. Element content of Ru-H_xWO_3 NN after HER test by XPS analysis.

Content / Sample after test	W /at.%	O /at.%	Ru /at.%	Ru /wt.%
**Raw**	13.7	58.61	0.42	1.21
**0.5 M H_2SO_4**	13.3	59.25	0.39	1.11
**1 M PBS**	13.6	54.69	0.41	1.18
**1 M KOH**	13.0	58.11	0.43	1.25

Supplementary Table S5. Fitted data of Nyquist plots in 0.5 M H_2SO_4.

Catalysts	η /mV	R_s /Ω	CPE_1 /F	R_{ct} /Ω	$C_φ$ /F	R_i /Ω	CPE_2 /F	R_c /Ω
Ru-H_xWO_3 NN	10	0.674	0.00277	0.1014	0.652	3.57	0.0513	0.441
	20	0.677	0.00697	0.0573	0.877	2.18	0.0506	0.381
	30	0.674	0.00385	0.0441	1.40	1.58	0.0403	0.399
	40	0.677	0.00307	0.0133	1.79	1.26	0.0372	0.379
	50	0.682	0.00279	0.0095	2.57	0.800	0.0294	0.386
	60	0.672	0.00383	0.080	4.34	0.326	0.0260	0.404
Ru-WO_3 NN	10	1.52	0.00527	0.198	0.0573	8.92	0.195	3.32
	20	1.51	0.00397	0.132	0.0727	6.20	0.141	2.97
	30	1.50	0.00913	0.0880	0.2555	4.83	0.0546	3.26
	40	1.52	0.00820	0.0755	0.6619	2.91	0.0500	2.83
	50	1.51	0.00886	0.0342	0.9282	1.74	0.0449	2.52
	60	1.50	0.00821	0.0170	1.12	0.772	0.0384	2.25
H_xWO_3 NN	10	0.905	0.00786	6.36	0.121	80.4	0.0160	59.1
	20	0.900	0.00742	6.12	0.126	78.2	0.0153	58.2
	30	0.903	0.00749	5.97	0.127	75.2	0.0146	65.2
	40	0.904	0.00737	5.62	0.131	71.9	0.0142	51.0
	50	0.901	0.00670	4.17	0.148	64.5	0.0137	52.9
	60	0.908	0.00671	3.91	0.166	53.5	0.0203	48.5
WO_3 NN	10	0.593	0.00172	6.59	0.00762	81.6	0.0159	76.0
	20	0.592	0.00174	6.32	0.00841	75.6	0.0153	73.6
	30	0.598	0.00187	5.95	0.0166	70.2	0.0150	68.9
	40	0.590	0.00181	4.90	0.0278	65.9	0.0110	71.0
	50	0.595	0.00177	4.16	0.0395	60.6	0.00792	64.1
	60	0.597	0.00170	3.04	0.0477	58.1	0.00701	62.9

Supplementary Table S6. Fitted data of Nyquist plots in 1 M PBS.

Catalysts	η /mV	R_s /Ω	CPE_1 /F	R_{ct} /Ω	$C_φ$ /F	R_i /Ω	CPE_2 /F	R_c /Ω
$Ru\text{-}H_xWO_3$ NN	10	1.88	0.00552	0.366	0.231	7.68	0.0253	0.678
	20	1.89	0.00556	0.3174	0.407	7.41	0.0318	0.643
	30	1.89	0.00567	0.3043	0.709	7.35	0.0259	0.692
	40	1.88	0.00481	0.2853	1.56	6.69	0.0218	0.652
	50	1.88	0.00489	0.2744	2.25	6.51	0.0209	0.686
	60	1.88	0.00512	0.2568	3.05	2.12	0.0181	0.630
$Ru\text{-}WO_3$ NN	10	2.20	0.0372	3.04	0.0497	9.42	0.487	6.15
	20	2.02	0.0302	2.87	0.0707	8.84	0.408	6.01
	30	2.08	0.0258	2.22	0.156	6.94	0.346	6.45
	40	2.10	0.0276	2.10	0.403	6.87	0.0998	7.84
	50	2.03	0.0265	1.59	0.719	6.41	0.0821	7.25
	60	2.08	0.0195	0.563	1.014	4.95	0.0608	6.95
H_xWO_3 NN	10	1.90	0.00501	5.22	0.105	78.7	0.0371	53.3
	20	1.89	0.00494	5.03	0.106	71.0	0.0307	49.7
	30	1.90	0.00720	4.13	0.110	65.4	0.0302	50.1
	40	1.94	0.00640	3.59	0.112	58.5	0.0282	44.6
	50	1.93	0.00462	3.66	0.119	47.3	0.0282	40.6
	60	1.96	0.00788	3.01	0.142	41.1	0.0280	31.1
WO_3 NN	10	1.92	5.16	5.16	0.00727	41.0	0.0252	75.9
	20	1.94	5.01	5.01	0.0840	39.6	0.0186	78.5
	30	1.94	4.58	4.58	0.0125	34.6	0.0179	70.3
	40	1.94	4.16	4.16	0.0181	32.3	0.0172	70.6
	50	1.95	3.16	3.16	0.0208	28.8	0.0167	68.7
	60	1.94	3.06	3.06	0.0257	23.3	0.0109	68.6

Supplementary Table S7 Fitted data of Nyquist plots in 1 M KOH.

Catalysts	η /mV	R_s /Ω	CPE_1 /F	R_{ct} /Ω	$C_φ$ /F	R_i /Ω	CPE_2 /F	R_c /Ω
Ru-H_xWO_3 NN	10	0.585	0.00433	0.170	0.302	7.27	0.0821	0.578
	20	0.572	0.00156	0.155	0.554	4.96	0.0651	0.687
	30	0.590	0.00540	0.139	1.05	2.94	0.0610	0.709
	40	0.583	0.00313	0.110	1.64	0.911	0.0524	0.699
	50	0.588	0.00220	0.0818	2.30	0.491	0.0423	0.668
	60	0.578	0.00358	0.0666	3.14	0.190	0.0433	0.699
Ru-WO_3 NN	10	0.675	0.0220	0.210	0.0501	8.83	0.168	3.30
	20	0.665	0.0158	0.201	0.0729	6.82	0.162	3.36
	30	0.603	0.0137	0.128	0.164	4.73	0.163	3.27
	40	0.647	0.0137	0.104	0.513	2.69	0.0955	3.57
	50	0.626	0.0124	0.095	0.820	1.71	0.0893	3.62
	60	0.633	0.0106	0.063	1.10	0.952	0.0773	3.97
H_xWO_3 NN	10	0.513	0.00224	7.63	0.0928	80.2	0.0108	68.8
	20	0.509	0.00242	7.35	0.103	79.6	0.00840	62.2
	30	0.512	0.00228	7.30	0.105	79.4	0.00628	69.1
	40	0.513	0.00251	7.20	0.113	69.6	0.00657	69.5
	50	0.517	0.00249	7.15	0.121	62.5	0.00625	67.4
	60	0.523	0.00209	7.05	0.136	55.6	0.00573	60.4
WO_3 NN	10	0.752	0.00503	7.24	0.00635	81.1	0.0396	80.7
	20	0.754	0.00525	7.13	0.0106	78.2	0.0231	79.7
	30	0.758	0.00555	7.01	0.0127	76.9	0.0163	81.6
	40	0.760	0.00565	6.91	0.0219	73.4	0.0141	79.5
	50	0.760	0.00552	6.25	0.0311	69.5	0.0125	77.4
	60	0.763	0.00561	5.92	0.0425	55.7	0.0111	76.4

References

1. Yokoyama, Y., Miyazaki, K., Miyahara, Y., Fukutsuka, T. & Abe, T. In Situ Measurement of Local pH at Working Electrodes in Neutral pH Solutions by the Rotating Ring-Disk Electrode Technique. *ChemElectroChem* **6**, 4750-4756 (2019).
2. Kresse, G. & Furthmüller, J. Efficiency of ab-initio total energy calculations for metals and semiconductors using a plane-wave basis set. *Comput. Mater. Sci.* **6**, 15-50 (1996).
3. Hammer, B., Hansen, L. B. & Nørskov, J. K. Improved adsorption energetics within density-functional theory using revised Perdew-Burke-Ernzerhof functionals. *Phys. Rev. B* **59**, 7413-7421 (1999).
4. Grimme, S., Ehrlich, S. & Goerigk, L. Effect of the damping function in dispersion corrected density functional theory. *J. Comput. Chem.* **32**, 1456-1465 (2011).
5. Wang, V., Xu, N., Liu, J.-C., Tang, G. & Geng, W.-T. VASPKIT: A user-friendly interface facilitating high-throughput computing and analysis using VASP code. *Comput. Phys. Commun.* **267**, 108033 (2021).
6. Henkelman, G., Uberuaga, B. P. & Jónsson, H. A climbing image nudged elastic band method for finding saddle points and minimum energy paths. *J. Chem. Phys.* **113**, 9901-9904 (2000).
7. Henkelman, G. & Jónsson, H. Improved tangent estimate in the nudged elastic band method for finding minimum energy paths and saddle points. *J. Chem. Phys.* **113**, 9978-9985 (2000).
8. Zheng, T. *et al.* Conductive Tungsten Oxide Nanosheets for Highly Efficient Hydrogen Evolution. *Nano Lett.* **17**, 7968-7973 (2017).
9. Spencer, M. A., Holzapfel, N. P., You, K.-E., Mpourmpakis, G. & Augustyn, V. Participation of electrochemically inserted protons in the hydrogen evolution reaction on tungsten oxides. *Chem. Sci.* **15**, 5385-5402 (2024).
10. Bunău, O. & Joly, Y. Self-consistent aspects of x-ray absorption calculations. *Journal of Physics: Condensed Matter* **21**, 345501 (2009).
11. Sun, Y. *et al.* Modulating electronic structure of metal-organic frameworks by introducing atomically dispersed Ru for efficient hydrogen evolution. *Nat. Commun.* **12**, 1369 (2021).
12. Zhao, Z. *et al.* Engineering active and robust alloy-based electrocatalyst by rapid Joule-heating toward ampere-level hydrogen evolution. *Nat. Commun.* **15**, 7475 (2024).
13. Yang, H. *et al.* Sequential Phase Conversion-Induced Phosphides Heteronanorod Arrays for Superior Hydrogen Evolution Performance to Pt in Wide pH Media. *Adv. Mater.* **34**, 2107548 (2022).
14. Li, J. *et al.* A fundamental viewpoint on the hydrogen spillover phenomenon of electrocatalytic hydrogen evolution. *Nat. Commun.* **12**, 3502 (2021).
15. Wang, C. *et al.* Octahedral Nanocrystals of Ru-Doped PtFeNiCuW/CNTs High-Entropy Alloy: High Performance Toward pH‐Universal Hydrogen Evolution Reaction. *Adv. Mater.* **36**, 2400433 (2024).
16. Zhang, F. *et al.* RuCo alloy bimodal nanoparticles embedded in N-doped carbon: a superior pH-universal electrocatalyst outperforms benchmark Pt for the hydrogen evolution reaction. *J. Mater. Chem. A* **8**, 12810-12820 (2020).
17. Shao, W. *et al.* Bioinspired Proton Pump on Ferroelectric HfO_2-Coupled Ir Catalysts with Bidirectional Hydrogen Spillover for pH-Universal and Superior Hydrogen Production. *J. Am. Chem. Soc.* **146**, 27486-27498 (2024).

18 Xing, M. *et al.* Amorphous/Crystalline $Rh(OH)_3$/CoP Heterostructure with Hydrophilicity/ Aerophobicity Feature for All-pH Hydrogen Evolution Reactions. *Adv. Energy Mater.* **13**, 2302376 (2023).

19 Wang, D. *et al.* Nickel metaphosphate supported ruthenium for all pH hydrogen evolution: From single atom, cluster to nanoparticle. *Appl. Catal., B* **325**, 122331 (2023).

20 Zhu, T. *et al.* Amorphous Ruthenium–Selenium Nanoparticles as a pH-Universal Catalyst for Enhanced Hydrogen Evolution Reaction. *ACS Catal.* **14**, 1914-1921 (2024).

21 Han, C. *et al.* Atomic Ru coordinated by channel ammonia in V-doped tungsten bronze for highly efficient hydrogen-evolution reaction. *Chin. J. Catal.* **51**, 80-89 (2023).

22 Kou, Z. *et al.* Electronic structure optimization of metal–phthalocyanine via confining atomic Ru for all-pH hydrogen evolution. *Energy Environ. Sci.* **17**, 1540-1548 (2024).

23 Chen, Y. *et al.* Intensifying the Supported Ruthenium Metallic Bond to Boost the Interfacial Hydrogen Spillover Toward pH‐Universal Hydrogen Evolution Catalysis. *Adv. Funct. Mater.* **34**, 2401452 (2024).

24 Song, H. *et al.* Single Atom Ruthenium-Doped CoP/CDs Nanosheets via Splicing of Carbon-Dots for Robust Hydrogen Production. *Angew. Chem., Int. Ed.* **60**, 7234-7244 (2021).

25 Zhang, H. *et al.* Constructing CoP/Ni_2P Heterostructure Confined Ru Sub-Nanoclusters for Enhanced Water Splitting in Wide pH Conditions. *Adv. Sci.* **11**, 2401398 (2024).

26 Zhang, Y. *et al.* Hetero‐Interface Manipulation in MoO_x@Ru to Evoke Industrial Hydrogen Production Performance with Current Density of 4000 mA cm^{-2}. *Adv. Energy Mater.* **13**, 2301492 (2023).

27 Xiao, X. *et al.* In Situ Growth of Ru Nanoparticles on (Fe,Ni)$(OH)_2$ to Boost Hydrogen Evolution Activity at High Current Density in Alkaline Media. *Small Methods* **4**, 1900796 (2020).

28 Du, H. *et al.* Cascade Reaction Enables Heterointerfaces-Enriched Nanoarrays for Ampere-Level Hydrogen Production. *Angew. Chem., Int. Ed.*, e202422393 (2024).

29 Zhang, J. *et al.* Surface-Reconstructed $CdNNi_3$ Antiperovskite Electrocatalyst: Unlocking Ampere-Level Current Density for Hydrogen Evolution. *ACS Nano* **18**, 32077-32087 (2024).

30 Yao, R. *et al.* Stable hydrogen evolution reaction at high current densities via designing the Ni single atoms and Ru nanoparticles linked by carbon bridges. *Nat. Commun.* **15**, 2218 (2024).

31 Liu, X. *et al.* In Situ-Grown Cobalt–Iron Phosphide-Based Integrated Electrode for Long-Term Water Splitting under a Large Current Density at the Industrial Electrolysis Temperature. *ACS Sustainable Chem. Eng.* **8**, 17828-17838 (2020).

32 Xie, L. *et al.* Flexible tungsten disulfide superstructure engineering for efficient alkaline hydrogen evolution in anion exchange membrane water electrolysers. *Nat. Commun.* **15**, 5702 (2024).

33 Wang, Y. *et al.* Industrially promising IrNi-FeNi3 hybrid nanosheets for overall water splitting catalysis at large current density. *Appl. Catal., B* **286**, 119881 (2021).

34 Hao, W. *et al.* Fabrication of practical catalytic electrodes using insulating and eco-friendly substrates for overall water splitting. *Energy Environ. Sci.* **13**, 102-110 (2020).

35 Zhao, R. *et al.* Tailoring a local acidic microenvironment on amorphous NiMoB catalyst to boost alkaline and neutral hydrogen evolution reactions. *Appl. Catal., B* **365**, 124928 (2025).

36 Deng, K. *et al.* Hydrogen spillover effect tuning the rate-determining step of hydrogen evolution over Pd/Ir hetero-metallene for industry-level current density. *Appl. Catal., B* **352**, 124047 (2024).
37 Da, Y. *et al.* Development of a Novel Pt_3V Alloy Electrocatalyst for Highly Efficient and Durable Industrial Hydrogen Evolution Reaction in Acid Environment. *Adv. Energy Mater.* **13**, 2300127 (2023).
38 Liu, R. *et al.* Design of Aligned Porous Carbon Films with Single‐Atom Co-N-C Sites for High-Current-Density Hydrogen Generation. *Adv. Mater.* **33**, 2103533 (2021).
39 Zheng, Z. *et al.* Boosting hydrogen evolution on MoS_2 via co-confining selenium in surface and cobalt in inner layer. *Nat. Commun.* **11**, 3315 (2020).
40 Zhang, Y. *et al.* Observation of a robust and active catalyst for hydrogen evolution under high current densities. *Nat. Commun.* **13**, 7784 (2022).
