# Supplementary material for: Lattice-hydrogen cycling mechanism enables pH-universal hydrogen evolution at ampere-level current densities
Source: Nat Commun. 2025 Dec 3;16:10863. doi: 10.1038/s41467-025-65909-3 (PMC12675675; doi:10.1038/s41467-025-65909-3)
Supplement: Supplementary file 2 — Description of Additional Supplementary Files [file 41467_2025_65909_MOESM2_ESM.pdf]

Description of Additional Supplementary Files

Supplementary Data 1: DFT calculation structure of Ru19-HxWO3
